# Variance Component Selection With Applications to Microbiome Taxonomic Data

**DOI:** 10.3389/fmicb.2018.00509

**Published:** 2018-03-28

**Authors:** Jing Zhai, Juhyun Kim, Kenneth S. Knox, Homer L. Twigg, Hua Zhou, Jin J. Zhou

**Affiliations:** ^1^Department of Epidemiology and Biostatistics, University of Arizona, Tucson, AZ, United States; ^2^Department of Biostatistics, University of California, Los Angeles, Los Angeles, CA, United States; ^3^Division of Pulmonary, Allergy, Critical Care, and Sleep Medicine, Department of Medicine, University of Arizona, Tucson, AZ, United States; ^4^Division of Pulmonary, Critical Care, Sleep, and Occupational Medicine, Indiana University Medical Center, Indianapolis, IN, United States

**Keywords:** Human Immunodeficiency Virus (HIV), lasso, longitudinal study, lung microbiome, MM-algorithm, variance component models, variable selection

## Abstract

High-throughput sequencing technology has enabled population-based studies of the role of the human microbiome in disease etiology and exposure response. Microbiome data are summarized as counts or composition of the bacterial taxa at different taxonomic levels. An important problem is to identify the bacterial taxa that are associated with a response. One method is to test the association of specific taxon with phenotypes in a linear mixed effect model, which incorporates phylogenetic information among bacterial communities. Another type of approaches consider all taxa in a joint model and achieves selection via penalization method, which ignores phylogenetic information. In this paper, we consider regression analysis by treating bacterial taxa at different level as multiple random effects. For each taxon, a kernel matrix is calculated based on distance measures in the phylogenetic tree and acts as one variance component in the joint model. Then taxonomic selection is achieved by the lasso (least absolute shrinkage and selection operator) penalty on variance components. Our method integrates biological information into the variable selection problem and greatly improves selection accuracies. Simulation studies demonstrate the superiority of our methods versus existing methods, for example, group-lasso. Finally, we apply our method to a longitudinal microbiome study of Human Immunodeficiency Virus (HIV) infected patients. We implement our method using the high performance computing language Julia. Software and detailed documentation are freely available at https://github.com/JingZhai63/VCselection.

## 1. Introduction

The advent of high-throughput sequencing technologies has produced extensive microbial community data, which reveals the impact of human microbes on health and various diseases (Mardis, 2008; Haas et al., 2011; Hodkinson and Grice, 2015; Kuleshov et al., 2016; Wang and Jia, 2016). Microbial community data collected from oral, skin, and gastrointestinal tract samples have received early attention (Eckburg et al., 2005; Gill et al., 2006; Turnbaugh et al., 2009; Dewhirst et al., 2010; Grice and Segre, 2011). Studies of the respiratory tract microbiome did not start until the discovery of microbiome in the lungs of both healthy (Erb-Downward et al., 2011; Morris et al., 2013; Twigg III et al., 2013) and diseased populations (Zemanick et al., 2011; Lozupone et al., 2013) using culture-independent techniques. A pulmonary microbiome dataset was sampled longitudinally from 30 HIV-infected individuals after starting highly active antiretroviral therapy (HAART). The objective is to study how the pulmonary microbiome impacts lung function of advanced HIV patients after HAART (Garcia et al., 2013; Lozupone et al., 2013; Twigg III et al., 2016).

After microbiome sequences have been acquired, they are usually clustered into Operational Taxonomic Units (OTUs): groups of sequences that correspond to taxonomic clusters or monophyletic groups (Caporaso et al., 2010). The abundance of an OTU is defined as the number of sequences in that OTU. The microbial community is then described by a list of OTUs, their abundances, and a phylogenetic tree. Regression methods have been a powerful tool to identify clusters of OTUs that are associated with or predictive of host phenotypes (Zhao et al., 2015; Wang and Zhao, 2016; Wang et al., 2017). Microbiome data presents several challenges. First microbiome abundances are sparse and the number of OTUs is usually much bigger than sample size. In our longitudinal data set, there are 2,964 OTUs and only two of them have abundance greater than 5%. When OTUs are included as predictors for clinical phenotypes in a regression model, regularizations are often used to overcome ill-conditioning. For example, Lin et al. (2014) proposed a linear log-contrast model with ℓ_1_ regularization. Another possible strategy to overcome the sparsity of microbial data is to cluster multiple OTUs into their higher phylogenetic levels, e.g., genus, order, and phylum. Shi et al. (2016) extended Lin et al.'s (2014) model to allow selecting taxa at different higher taxonomic ranks. However, both methods overlook the distance information in the phylogenetic tree. A network-constrained sparse regression is proposed to achieve better prediction performance through a Laplacian regularization (Chen et al., 2012b, 2015b). Another popular approach for sparse linear regression is the group-wise selection scheme, group-lasso, which selects an entire group for inclusion or exclusion (Yuan and Lin, 2006; Garcia et al., 2013; Simon et al., 2013; Yang and Zou, 2015). Therefore, group-lasso is a natural tool for incorporating group information defined by the phylogenetic tree, but still misses fine level information. To encourage hierarchically close species to have similar effects on the phenotype, Wang and Zhao (2016) and Wang et al. (2017) both used tree topology information and fused variables that stay closer in a tree. However, this assumption may be violated. For example, the bacteria *Clostridia*, some species in this class convert dietary fiber into anti-inflammatory short-chain fatty acids, while others cause severe colitis. We, therefore, need a method that can incorporate biologically meaningful cluster information, phylogenetic distance, or tree information, can encourage sparse feature selection, and can handle possible adverse effect within clusters.

By modeling microbiome cluster effects as random effects, Zhai et al. (2017b) proposed a variance component model

(1) $$\begin{array}{l} y=X\beta +Zb+\sum_{l}^{L}h_{l}+\varepsilon \\ b\sim N(0,\sigma_{d}^{2}I_{n}),h_{l}\sim N(0,\sigma_{gl}^{2}K_{l}),\varepsilon \sim N(0,\sigma_{e}^{2}I_{n}), \end{array}$$

where ***y***, ***X***, and **ε** are the vertically stacked vectors/matrices of ***y***_*i*_, ***X***_*i*_, and **ε**_*i*_. The ***y***_*i*_ is an *n*_*i*_ × 1 vector of *n*_*i*_ repeated measures of the quantitative phenotype for an individual *i*. ***X***_*i*_ is the *n*_*i*_ × *p* covariates. The **ε**_*i*_ is an *n*_*i*_ × 1 vector of the random error. $Z_{i}={\left(1,\ldots ,1\right)}^{\prime }$ is an *n*_*i*_ × 1 design matrix linking the vector of random effects *b*_*i*_ to ***y***_*i*_. ***Z*** is a block diagonal matrix with ***Z***_*i*_ on its diagonal. **β** is a *p* × 1 vector of fixed effects. The ***b*** = (*b*_*i*_) is the subject-specific random effects. *L* is the total number of microbiome taxonomic clusters, *N* is the total number of individuals and $\overset{N}{\underset{i=1}{\sum }}n_{i}$ is the total number of observations. In model (Equation 1), *h*_*l*_ is the random effects generated by microbiome taxa *l* with covariance $\sigma_{gl}^{2}K_{l}$. ***K***_*l*_ is a positive-definite kernel matrix derived from a distance matrix that is calculated based on the OTU abundances of taxa in the phylogenetic tree. Two common distance matrices are UniFrac Distance (Lozupone and Knight, 2005) and Bray-Curtis dissimilarity (Bray and Curtis, 1957). Therefore,

(2) $$\text{Var}(y)=\sigma_{d}^{2}Z^{\prime }Z+\sum_{l=1}^{L}\sigma_{g_{l}}^{2}K_{l}+\sigma_{e}^{2}I_{n},$$

where $\sigma_{g_{l}}^{2}$ and $\sigma_{d}^{2}$ are the phenotypic variance from microbiome clusters and between subject variance from repeated measurements. $\sigma_{e}^{2}$ is the within-subject variance that cannot be explained by either microbiome or repeated measurements. To identify associated microbiome taxa at different phylogeny levels is to select non-zero variance components at different phylogeny levels.

In this article, we adopt a penalized likelihood approach by regularizing variance components based on linear mixed effect models: variance component lasso selection (VC-lasso). We incorporate the phylogenetic tree information by using kernel matrices. We reduce the dimensionality of large and very sparse OTU abundances within a cluster by translating them into a random effect. Furthermore, our method can be applied to a longitudinal design, where an unpenalized variance component that captures the correlation of repeated measurements is included. Our Majorization-Minimization (MM) algorithm for variance component selection guarantees estimation and selection computational efficiency (Hunter and Lange, 2004; Hunter and Li, 2005; Zhou et al., 2011, 2015; Lange, 2016). Many statistical methods have been proposed related to the selection of random effects. Ibrahim et al. (2011) considered jointly selecting fixed and random effect in mixed effect model using the maximum likelihood with the smoothly clipped absolute deviation (SCAD) and adaptive lasso penalization. Fan and Li (2012) proposed a group variable selection strategy to select and estimate important random effects. Hui et al. (2017) extended this strategy to generalized linear mixed model by combining the penalized quasi-likelihood (PQL) estimation with sparsity-inducing penalties on the fixed and random coefficients. However, none of these methods can be easily extended to microbiome data and none of them use variance component regularization.

The rest of this paper is organized as follows. We introduce the variance component lasso selection method in section 2. Section 3 conducts comparative simulation studies. Section 4 presents simulation and real data analysis results. We conclude with a discussion in section 5.

## 2. Methods

### 2.1. Lasso penalized log-likelihood

We consider model (Equation 2) with model parameters **β** and $\sigma^{2}=\left(\sigma_{1}^{2},\ldots ,\sigma_{m}^{2}\right)$. The log-likelihood of our model is:

(3) $$L(\beta ,\sigma^{2};y,X)=-\frac{1}{2}\text{ln det}(V)-\frac{1}{2}(Y-X\beta {)}^{\prime }V^{-1}(Y-X\beta ),$$

where

$$V=\sum_{i=1}^{m}\sigma_{i}^{2}V_{i}.$$

For the selection of non-zero variance components among a large number of variance components, we estimate the regression parameter **β** and **σ**^2^ by minimizing the lasso penalized log-likelihood function

(4) $$pl(\beta ,\sigma^{2};y,X,\lambda )=-L(\beta ,\sigma^{2})+\lambda \sum_{i=1}^{m}c_{i}\sigma_{i},$$

subject to nonnegativity constraint σ_*i*_ ≥ 0. The first part −*L*(**β**, **σ**^2^) of the penalized function (Equation 4) is the negative log-likelihood defined in Equation (3). The second part is the lasso penalty to enforce shrinkage of high-dimensional components. We do not penalize fixed effects **β**. λ is the tuning parameter controlling model complexity; *c*_*i*_ ∈ {0, 1} allows differential shrinkage of specific variance components. For example, when modeling longitudinal phenotypes with random intercept model, the corresponding variance component is unpenalized and always stays in the model. *c*_*i*_ can be chosen using different weighting schemes based on prior knowledge such as functional annotations.

### 2.2. Minimization of penalized likelihood via MM algorithm

Minimizing the penalized negative log-likelihood is challenging due to non-convexity. Based on the Majorization-Minimization (MM) algorithm (Lange et al., 2000; Hunter and Lange, 2004), Zhou et al. (2015) proposed a strategy for maximizing the log-likelihood Equation (3) by alternate updating **β** and variance components **σ**^2^. We follow the same strategy to solve the lasso penalized likelihood estimation problem (Algorithm 1).

**Algorithm 1:**
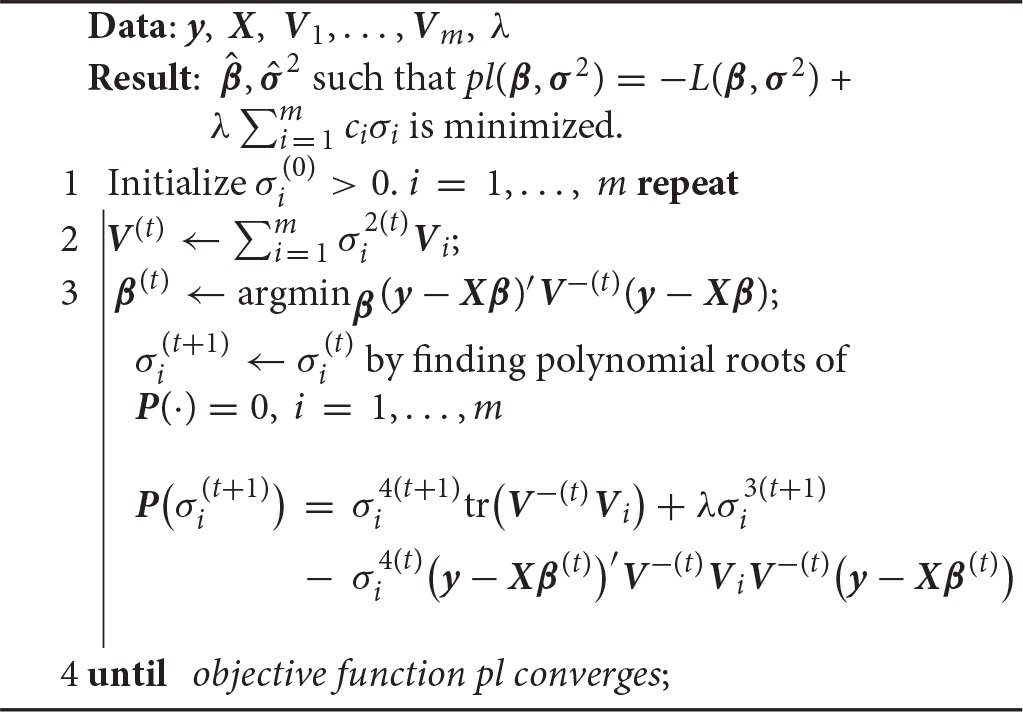
MM algorithm for minimizing lasso penalized likelihood (Equation 4).

Given **σ**^2(*t*)^, updating **β** is a general least squares problem with solution

(5) $$\beta^{\left(t+1\right)}={\left(X^{\prime }V^{-(t)}X\right)}^{-1}X^{\prime }V^{-(t)}y,$$

where ***V***^−(*t*)^ represents the *t*th-step update of ***V***^−1^. Given **β**^(*t*)^, updating the variance components **σ**^2^ invokes the MM principle. To minimize the objective function *pl*(**θ**), where **θ** = (**β**, **σ**^2^), the majorization step operates by creating a surrogate function *g*(**θ**|**θ**^(*t*)^) that satisfies two conditions

$$\begin{array}{l} \text{dominance condition }:pl(\theta )\leq g(\theta |\theta^{\left(t\right)})\text{ for all }\theta \\ \text{ tangent condition }:pl(\theta^{\left(t\right)})=g(\theta^{\left(t\right)}|\theta^{\left(t\right)}). \end{array}$$

The second M of the MM principle minimizes the surrogate function to produce the next iterate **θ**^(*t*+1)^. Then we have

$$pl(\theta^{\left(t+1\right)})\leq g(\theta^{\left(t+1\right)}|\theta^{\left(t\right)})\leq g(\theta^{\left(t\right)}|\theta^{\left(t\right)})=pl(\theta^{\left(t\right)}).$$

Therefore, when the surrogate function is minimized, the objective function *f*(**θ**) is driven downhill. We combine two following majorizations to construct the surrogate function. First, with all ***V***_*i*_ being positive semidefinite, Zhou et al. (2015) show that

$$\begin{array}{l} V^{\left(t\right)}V^{-1}V^{\left(t\right)}=\left(\sum_{i=1}^{m}\sigma_{i}^{2(t)}V_{i}\right){\left(\sum_{i=1}^{m}\sigma_{i}^{2}V_{i}\right)}^{-1}\left(\sum_{i=1}^{m}\sigma_{i}^{2(t)}V_{i}\right)\\ \;≼\sum_{i=1}^{m}\frac{\sigma_{i}^{2(t)}}{\sum_{j}\sigma_{j}^{2(t)}}\left(\frac{\sum_{j}\sigma_{j}^{2(t)}}{\sigma_{i}^{2(t)}}\sigma_{i}^{2(t)}V_{i}\right)\\ \;{\left(\frac{\sum_{j}\sigma_{j}^{2(t)}}{\sigma_{i}^{2(t)}}\sigma_{i}^{2}V_{i}\right)}^{-1}\left(\frac{\sum_{j}\sigma_{j}^{2(t)}}{\sigma_{i}^{2(t)}}\sigma_{i}^{2(t)}V_{i}\right)\\ \;=\sum_{i=1}^{m}\frac{\sigma_{i}^{4(t)}}{\sigma_{i}^{2}}V_{i}V_{i}^{-1}V_{i}=\sum_{i=1}^{m}\frac{\sigma_{i}^{4(t)}}{\sigma_{i}^{2}}V_{i}, \end{array}$$

leading to the first majorization

(6) $$\begin{array}{l} \;(y-X\beta {)}^{\prime }V^{-1}(y-X\beta )\\ ≼(y-X\beta {)}^{\prime }V^{-(t)}\left(\sum_{i=1}^{m}\frac{\sigma_{i}^{4(t)}}{\sigma_{i}^{2}}V_{i}\right)V^{-(t)}(y-X\beta ). \end{array}$$

It separates the variance components $\sigma_{1}^{2},\ldots ,\sigma_{m}^{2}$ in the quadratic term of the log-likelihood function (Equation 4). By the supporting hyperplane inequality, the second majorization is

(7) $$\text{ln det}V\leq \text{ln det}V^{\left(t\right)}+\text{tr}[V^{-(t)}(V-V^{\left(t\right)})],$$

which separates $\sigma_{1}^{2},\ldots ,\sigma_{m}^{2}$ in the log-determinant term of Equation (4). The overall majorization *g*(**σ**^2^|**σ**^2(*t*)^) of *pl*(**β**, **σ**^2^) is obtained by combining Equations (6) and (7)

(8) $$\begin{array}{l} g(\sigma^{2}|\sigma^{2(t)})=\frac{1}{2}\text{tr}(V^{-(t)}V)+\frac{1}{2}(y-X\beta^{\left(t\right)}{)}^{\prime }V^{-(t)}\\ \;\left(\sum_{i=1}^{m}\frac{\sigma_{i}^{4(t)}}{\sigma_{i}^{2}}V_{i}\right)V^{-(t)}(y-X\beta^{\left(t\right)})+\lambda \sum_{i=1}^{m}\sigma_{i}+s^{\left(t\right)}\\ \;=\sum_{i=1}^{m}[\frac{\sigma_{i}^{2}}{2}\text{tr}(V^{-(t)}V_{i})+\frac{\sigma_{i}^{4(t)}}{2\sigma_{i}^{2}}(y-X\beta^{\left(t\right)}{)}^{\prime }\\ \;V^{-(t)}V_{i}V^{-(t)}(y-X\beta^{\left(t\right)})+\lambda \sigma_{i}]+s^{\left(t\right)}, \end{array}$$

where *s*^(*t*)^ is an irrelevant constant term.

We minimize the surrogate function (Equation 8) by setting the derivative of *g*(**σ**^2^|**σ**^2(*t*)^) to zero. The update $\sigma_{i}^{\left(t+1\right)}$ for variance component $\sigma_{i}^{\left(t\right)}$ is chosen among the positive roots of the polynomial

$$\begin{array}{l} P(\sigma_{i}^{\left(t+1\right)})=\sigma_{i}^{4(t+1)}\text{tr}(V^{-(t)}V_{i})+\lambda \sigma_{i}^{3(t+1)}\\ \;-\;\sigma_{i}^{4(t)}(y-X\beta^{\left(t\right)}{)}^{\prime }V^{-(t)}V_{i}V^{-(t)}(y-X\beta^{\left(t\right)}) \end{array}$$

or 0, whichever yields the largest objective value. The alternating updates repeat until

$$|pl(\beta^{\left(t+1\right)},\sigma^{2(t+1)})-pl(\beta^{\left(t\right)},\sigma^{2(t)})|<tol*(|pl(\beta^{\left(t\right)},\sigma^{2(t)})|+\;1),$$

where *tol* is the pre-specified tolerance. The default tolerance is 10^−4^.

### 2.3. Tuning parameter selection

The tuning parameter λ in the penalized likelihood estimation is chosen by a 5-fold cross-validation procedure based on $g\text{-Measure}=\sqrt{\text{sensitivity}*\text{specificity}}$. *g*-Measure is an indicator of the model selection accuracy. *g*-Measure = 1 indicates the best accuracy and *g*-Measure = 0 the worst (Zhai et al., 2017a). It can counteract the imbalance between the number of of irrelevant and relevant clusters. Therefore, we present *g*-Measure instead of sensitivity (true positive rate) and specificity (true negative rate) alone (Supplementary Material section 3). Akaike Information Criterion (AIC) (Akaike, 1998) and Schwarzs Bayesian Information Criterion (BIC) (Schwarz et al., 1978) are used in the real data analysis. Performance comparisons between cross-validation and AIC/BIC are provided in the Supplementary Material section 4.

### 2.4. Software implementation

We implement our method using the high performance computing language Julia. UniFrac distance matrices are computed using our Julia package PhylogeneticDistance.

## 3. Simulation

In this section, we conduct simulation studies to evaluate the variable selection and prediction performance of VC-lasso and compare the results with the conventional method group-lasso as implemented in the gglasso package (Yang and Zou, 2015). Phenotypes are simulated based on one real pulmonary microbiome dataset and one simulated longitudinal microbiome dataset. We first describe real and simulated microbiome abundance data, phylogenetic tree, and then detail our four phenotype simulation schemes (Table 1).

**Table 1:** Simulation parameter configurations.

	**Non-zero variance components**	**Cluster/kernel**	**Design**	**$\sigma_{g}^{2}$^†^**	**Method**
**Scenario 1**: *Selection under different sample sizes*					
*n* = 20, 50, 100; simulated count data	*l* = 1, 2, 3, 4, 5	genus; ***K***_*W*_	longitudinal; cross-sectional	1, 5, 25, 100	VC-lasso group-lasso
**Scenario 2**: *Selection under different number of non-zero variance components*					
*n* = 50; simulated count data	(i) *l* = 20, 30; (ii) *l* = 1, 2, 3, 4, 5; (iii) *l* = 1, 2, 3, …, 15;	genus; ***K***_*W*_	longitudinal; cross-sectional	1, 5, 25, 100	VC-lasso group-lasso
**Scenario 3**: *Selection under different UniFrac distance kernels*					
*n* = 50; simulated count data	*l* = 1, 2, 3, 4, 5	genus; ***K***_*W*_, ***K***_*UW*_, ***K***_*VAW*_, ***K***_0_, ***K***_0.5_	longitudinal; cross-sectional	1, 5, 25, 100	VC-lasso group-lasso
**Scenario 4**: *Selection under fixed effect model*					
*n* = 50; simulated count data	*l* = 20, 30; *l* = 1, 2, 3, 4, 5; *l* = 1, 2, 3, …, 15;	genus; ***K***_*W*_	cross-sectional	1, 5, 25, 100	VC-lasso group-lasso

*Throughout simulations, $\sigma_{e}^{\text{2}}=\text{1}$, β_1_ = β_2_ = 0.1. We use $\sigma_{d}^{\text{2}}=\text{0.6}$ and 3 repeated measurements in the longitudinal design. We use $\sigma_{d}^{\text{2}}=\text{0}$ for the cross-sectional design. Group-lasso is performed only in the cross-sectional design*.

† *The non-zero variance components are assumed to have equal effect strength in each simulation setting*.

The real pulmonary microbiome data has been discussed in Twigg III et al. (2016). Thirty individuals were recruited. During up to three-years follow-up, lung functions and microbiome composition were measured 2–4 times for each individual. The longitudinal microbiome taxonomic data is summarized as 2,964 OTUs with a phylogenetic tree (Twigg III et al., 2016). Longitudinal microbiome abundance data is generated by a Zero-Inflated Beta Random Effect model using R package ZIBR in Supplementary Material section 2 (Chen and Li, 2016). For cross-sectional design, we generate taxonomic data using a Dirichlet-Multinomial (DM) model (Chen et al., 2012a). Simulation parameters, such as proportion of each OTU and the overall dispersion, are estimated from our real pulmonary microbiome abundance data.

Given simulated microbiome count data and taxonomic information, we classify 2,353 of 2,964 OTUs to 30 genera (taxa clusters) and the remaining 611 of 2,964 OTUs are grouped into the 31st cluster named *other* (Table 2). As described in Supplementary Material section 1, UniFrac distance matrices (***D***) of the 31 clusters are computed and converted to kernel matrices as

(9) $$K\;=-\frac{1}{2}(I-\frac{11^{\prime }}{n})D^{2}(I-\frac{11^{\prime }}{n})$$

followed by a positive definiteness correction (Chen and Li, 2013; Zhao et al., 2015). All of the microbiome kernel matrices ***K*** are scaled to have unit Frobenius norm.

**Table 2:** List of 31 Genera.

	**Genus**	**Phylum**	**No of OTU**	**Mean Reads**
1	*Actinomyces*	*Actinobacteria*	150	230.59
2	*Anaerococcus*	*Firmicutes*	17	2.90
3	*Atopobium*	*Actinobacteria*	22	40.83
4	*Campylobacter*	*Proteobacteria*	31	51.05
5	*Capnocytophaga*	*Bacteroidetes*	31	70.81
6	*Catonella*	*Firmicutes*	22	40.09
7	*Corynebacterium*	*Actinobacteria*	47	12.22
8	*Flavobacterium*	*Bacteroidetes*	25	5.08
9	*Fusobacterium*	*Fusobacteria*	55	174.29
10	*Gemella*	*Firmicutes*	17	72.11
11	*Lactobacillus*	*Firmicutes*	33	141.10
12	*Leptotrichia*	*Fusobacteria*	15	12.40
13	*Megasphaera*	*Firmicutes*	14	36.99
14	*Methylobacterium*	*Proteobacteria*	11	2.88
15	*Neisseria*	*Proteobacteria*	18	109.61
16	*OD1_genera_incertae_sedis*	*OD1*	75	0.92
17	*Parvimonas*	*Firmicutes*	20	76.46
18	*Peptoniphilus*	*Firmicutes*	11	1.16
19	*Porphyromonas*	*Bacteroidetes*	42	134.41
20	*Prevotella*	*Bacteroidetes*	304	833.35
21	*Rothia*	*Actinobacteria*	16	49.83
22	*Selenomonas*	*Firmicutes*	50	16.16
23	*Sneathia*	*Fusobacteria*	12	37.09
24	*Sphingomonas*	*Proteobacteria*	14	0.61
25	*SR1_genera_incertae_sedis*	*SR1*	17	5.95
26	*Streptococcus*	*Firmicutes*	66	1, 107.81
27	*TM7_genera_incertae_sedis*	*TM7*	61	40.54
28	*Treponema*	*Spirochaetes*	60	51.62
29	*Unclassified*	*Unclassified*^†^	1,068	258.65
30	*Veillonella*	*Firmicutes*	29	370.85
31	*Others*	*Others*	611	1, 009.88

*Summary of phylum information, the number of OTUs, and the average abundance (across sample and time points) within each genus from the pulmonary microbiome dataset are shown*.

† *The genus unclassified may belong to phylum unclassified or other 12 phyla*.

Phenotypes are simulated based on the following scenarios.

### 3.1. Scenario 1: selection under different sample size

Longitudinal and cross-sectional responses are generated by

(10) $$y\sim N(X_{1}\beta_{1}+X_{2}\beta_{2},\;\sigma_{d}^{2}ZZ^{\prime }+\sum_{l=1}^{L}\sigma_{gl}^{2}K_{l}+\sigma_{e}^{2}I),$$

where $\sigma_{g_{l}}^{2}>0$ for *l* = 1, …, 5 and $\sigma_{g_{l}}^{2}=0$ otherwise. The total number of variance components for microbiome clusters is *L* = 31. The true model has five non-zero variance components including *Anaerococcus, Atopobium, Actinomyces, Campylobacter*, and *Capnocytophaga*. We compare the selection performance at three sample sizes: *n* = 20, 50, 100. For cross-sectional design, responses are simulated by setting $\sigma_{d}^{2}=0$.

### 3.2. Scenario 2: selection under different numbers of non-zero variance components

The sample size is fixed at *n* = 50 in this scenario. Responses are generated by model (Equation 10) with different numbers of non-zero variance components. In Supplementary Material section 5, VC-lasso is evaluated when the number of variance components in the model is large.

1. 2 non-zero variance components: $\sigma_{g_{20}}^{2}>0$, $\sigma_{g_{30}}^{2}>0$, and $\sigma_{g_{l}}^{2}=0$ otherwise. Two associated genera are *prevolleta* and *veillonella*.
2. 5 non-zero variance components: $\sigma_{g_{l}}^{2}>0$ for *l* = 1, 2, …, 5 and $\sigma_{g_{l}}^{2}=0$ otherwise. Associated clusters are *Anaerococcus, Atopobium, Actinomyces, Campylobacter*, and *Capnocytophaga*.
3. 15 non-zero variance components: $\sigma_{g_{l}}^{2}>0$ for *l* = 1, 2, …, 15 and $\sigma_{g_{l}}^{2}=0$ otherwise. Associated clusters, including *Actinomyces, Anaerococcus*, …, and *Neisseria* are listed in Table 2.

### 3.3. Scenario 3: selection under different UniFrac distance kernels

The sample size is fixed at *n* = 50 with 5 non-zero variance components. We compare the selection performance using kernels defined by 5 different distance measures: variance adjusted weighted UniFrac distance (***K***_*VAW*_) Chang et al., 2011), generalized UniFrac distance (***K***_0_, ***K***_0.5_) (Chen et al., 2012a), unweighted UniFrac distance (***K***_*UW*_) (Lozupone and Knight, 2005), and weighted UniFrac distance (***K***_*W*_) (Lozupone et al., 2007).

### 3.4. Scenario 4: selection under fixed effect model

We again use the sample size *n* = 50 and vary the number of clusters containing signal. Responses are simulated by a fixed effect model

(11) $$\begin{array}{l} y\sim N(X_{1}\beta_{1}+X_{2}\beta_{2}+G_{1}^{*}\gamma_{1}+G_{2}^{*}\gamma_{2}+\ldots \\ +\;G_{u}^{*}\gamma_{u},\;\sigma_{e}^{2}I), \end{array}$$

where $G_{1}^{*},\;G_{2}^{*},\ldots ,G_{u}^{*}$ are OTU count matrices of different clusters scaled by their sample maximum. *u* is the total number of clusters with effects that ranges from 2 to 15. Fixed effect vector **γ**_*l*_ for cluster *l* are generated from $\gamma_{l}\sim N\left(0,\sigma_{gl}^{2}I\right)$ and are fixed for each simulation replicate.

We applied VC-lasso to scenarios 1-3 using both longitudinal and cross-sectional designs. Scenario 4 is performed using a cross-sectional design only. We compare our approach with group-lasso (R package gglasso) in all 4 scenarios for cross-sectional design because the gglasso package cannot handle longitudinal data.

We set the within-individual variance $\sigma_{e}^{2}=1$ throughout simulations. The between individual variance of random intercept is set to $\sigma_{d}^{2}=0.6$ for longitudinal design and $\sigma_{d}^{2}=0$ otherwise (Twigg III et al., 2016). The effect strength is set to $\sigma_{g}^{2}=1,5,25,100$ (Chen et al., 2015a). We set the non-zero variance components to have the same effect strength under each setting, therefore omit subscript *l*. Two covariates ***X***_1_ and ***X***_2_ are generated from the standard normal distribution and effect sizes are set to β_1_ = β_2_ = 0.1. 1000 Monte Carlo simulation replicates are generated. We split each dataset to training (80%) and testing (20%). Five-fold cross-validation is performed in training set to estimate the optimal λ^*^. Selection performance is evaluated and reported by applying λ^*^ to the testing set.

## 4. Results

### 4.1. Analysis of simulated data

**Figure 1:**
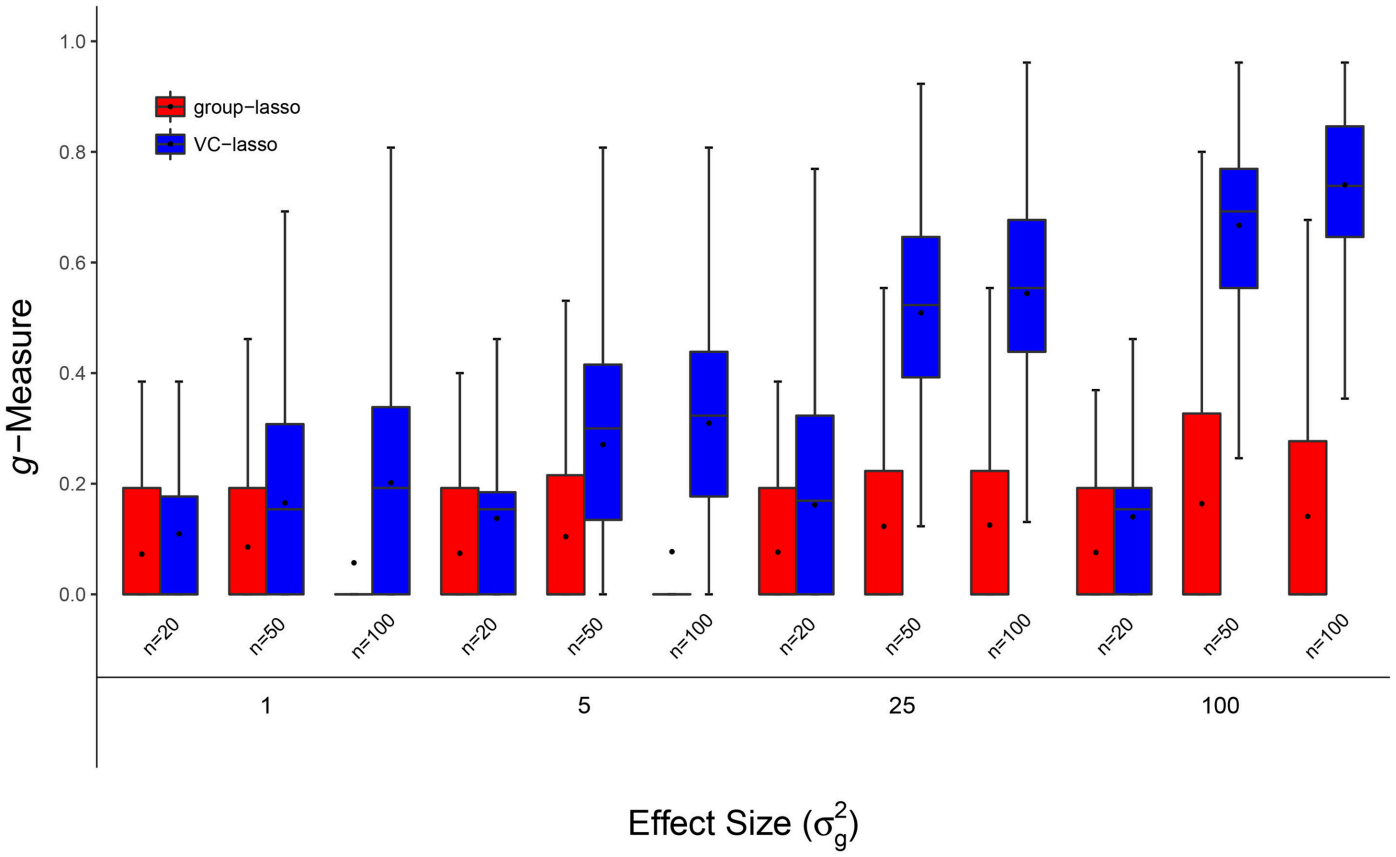
Scenario 1: Estimated ***g***-Measure of both VC-lasso and group-lasso under different sample sizes for models with 5 non-zero variance components in a cross-sectional design. Three sample sizes, *n* = 20, 50, 100, are compared and $\sigma_{d}^{2}=0$.

**Figure 2:**
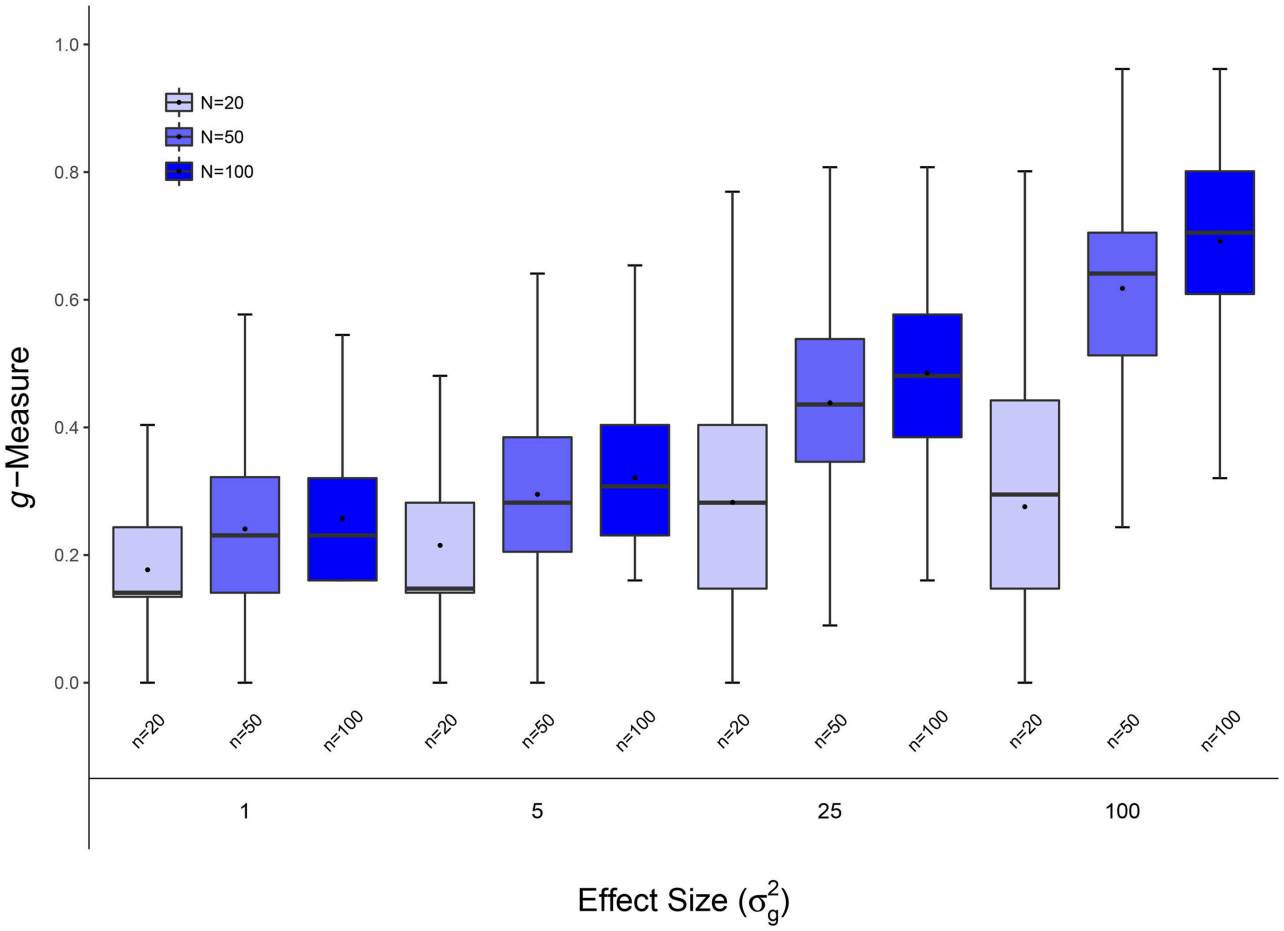
Scenario 1: Estimated ***g***-Measure of VC-lasso under different sample sizes for models with 5 non-zero variance components in a longitudinal design. Three sample sizes, *n* = 20, 50, 100, are compared and $\sigma_{d}^{2}=0.6$.

**Figure 3:**
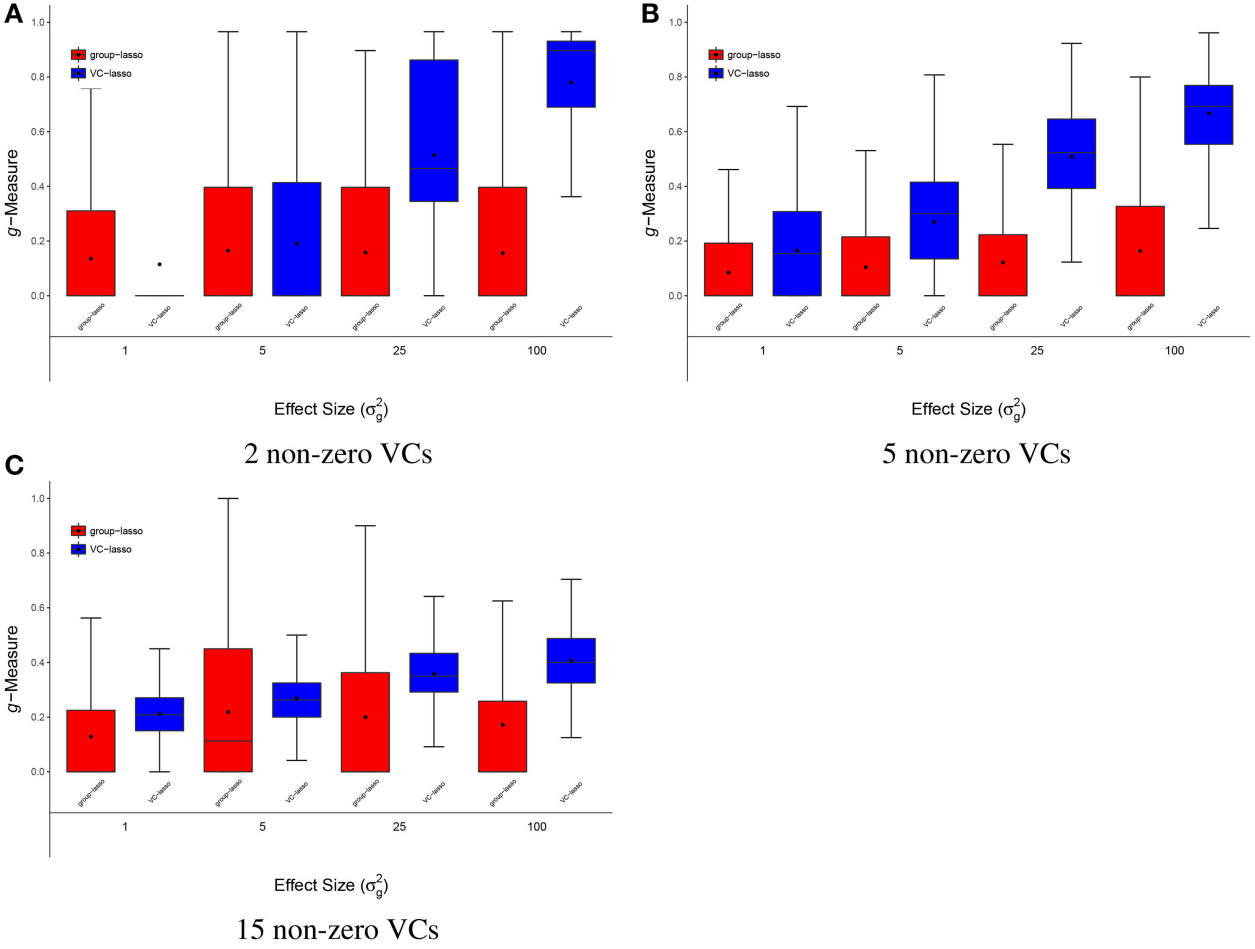
Scenario 2: Estimated ***g***-Measure of both VC-lasso and group-lasso under different number of non-zero variance components in a cross-sectional design. The number of non-zero variance components (VCs) are set to 2 **(A)**, 5 **(B)**, 15 **(C)**, sample size is *n* = 50, and $\sigma_{d}^{2}=0$.

**Figure 4:**
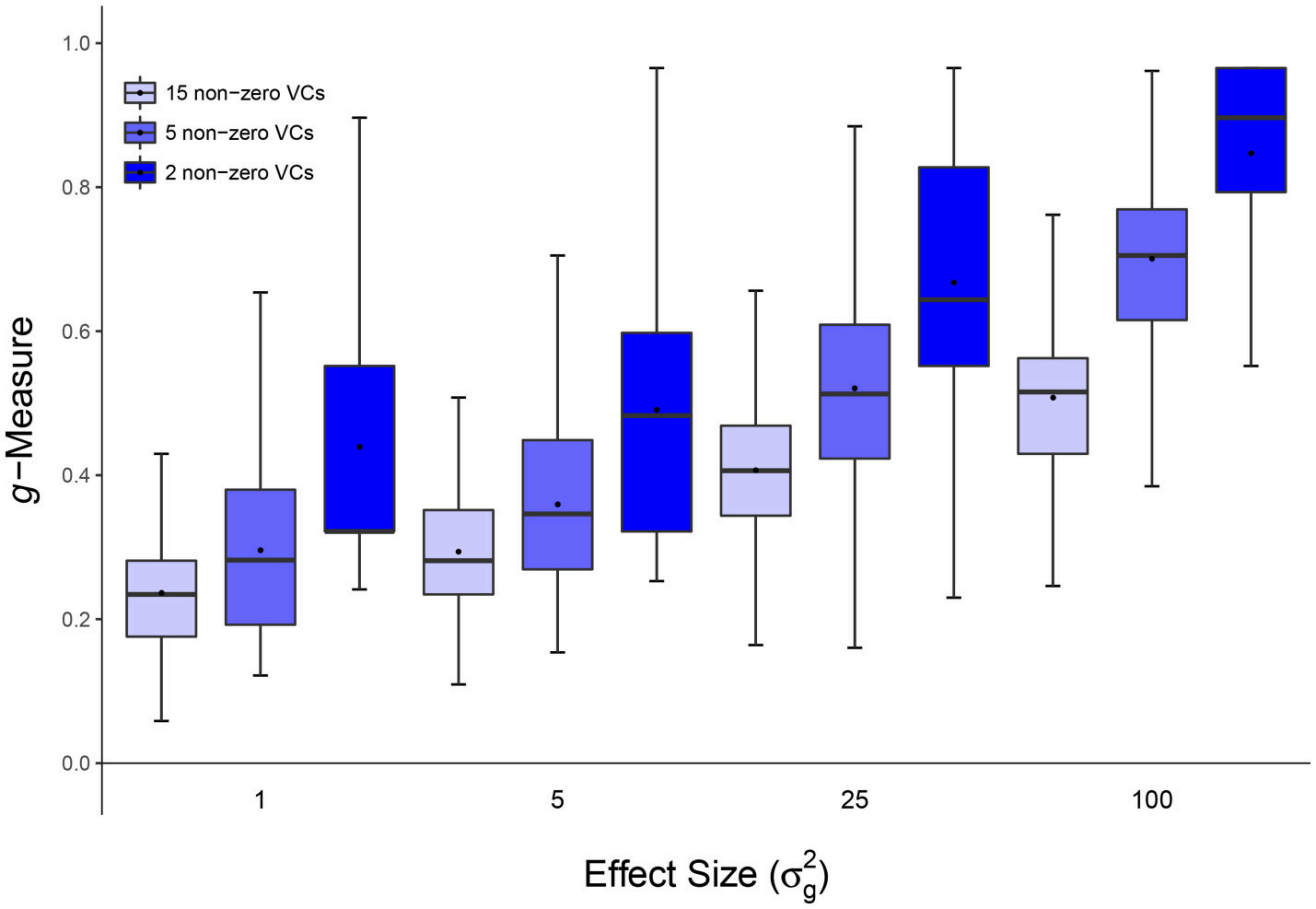
Scenario 2: Estimated ***g***-Measure of VC-lasso under different number of non-zero variance components in a longitudinal design. Three different numbers of non-zero variance components (VCs), 2, 5, 15, are shown, sample size is set to *n* = 50 and $\sigma_{d}^{2}=0.6$.

**Figure 5:**
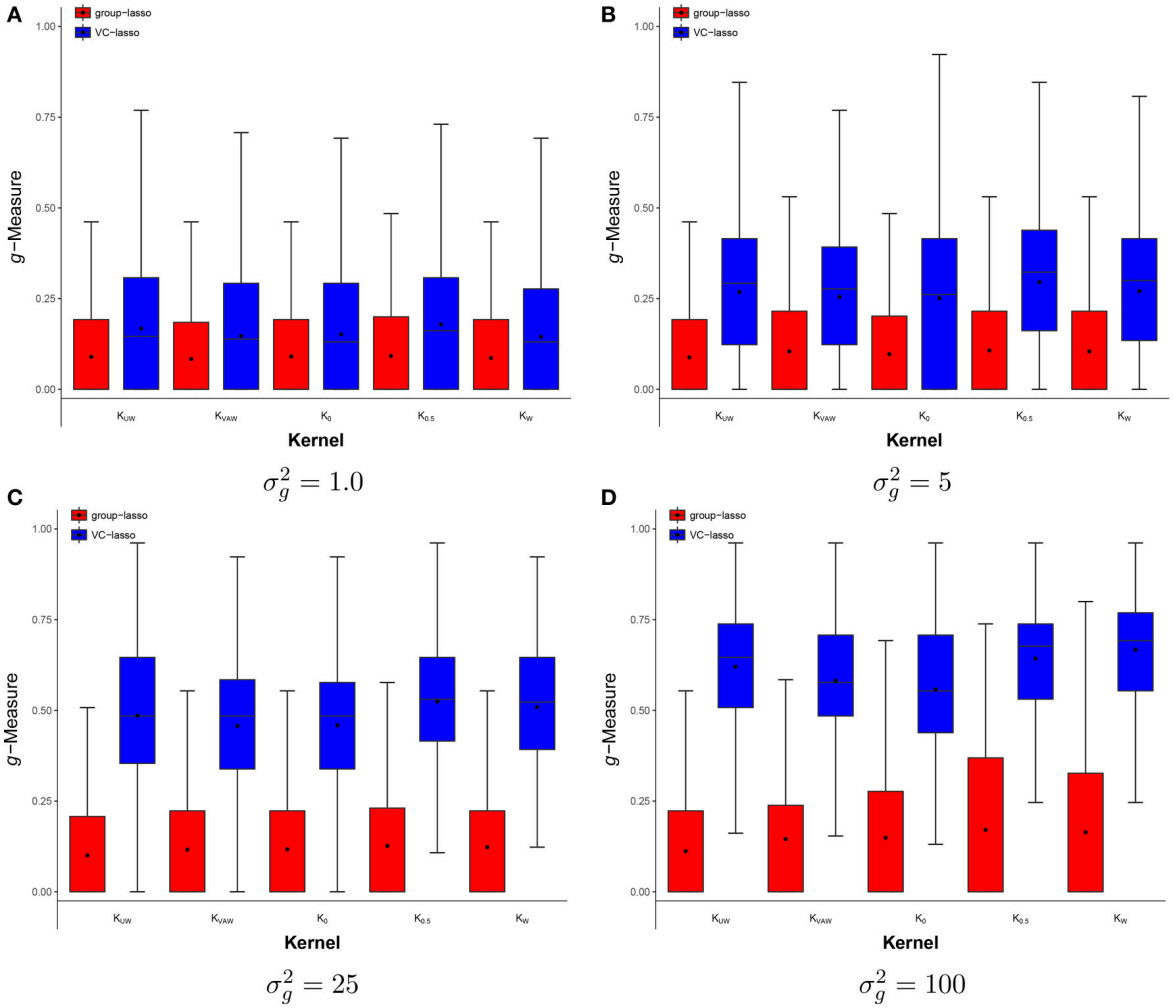
Scenario 3: Estimated ***g***-Measure of both VC-lasso and group-lasso under different UniFrac distance kernels in a cross-sectional design. Five different kernels, *K*_*UW*_, *K*_*VAW*_, *K*_0_, *K*_0.5_and*K*_*W*_, and two methods, VC-lasso and group-lasso, are displayed in a cross-sectional design. Four effect strengths, 1 **(A)**, 5 **(B)**, 25 **(C)**, and 100 **(D)** are shown. There are 5 non-zero variance components, sample size is *n* = 50, and $\sigma_{d}^{2}=0$.

**Figure 6:**
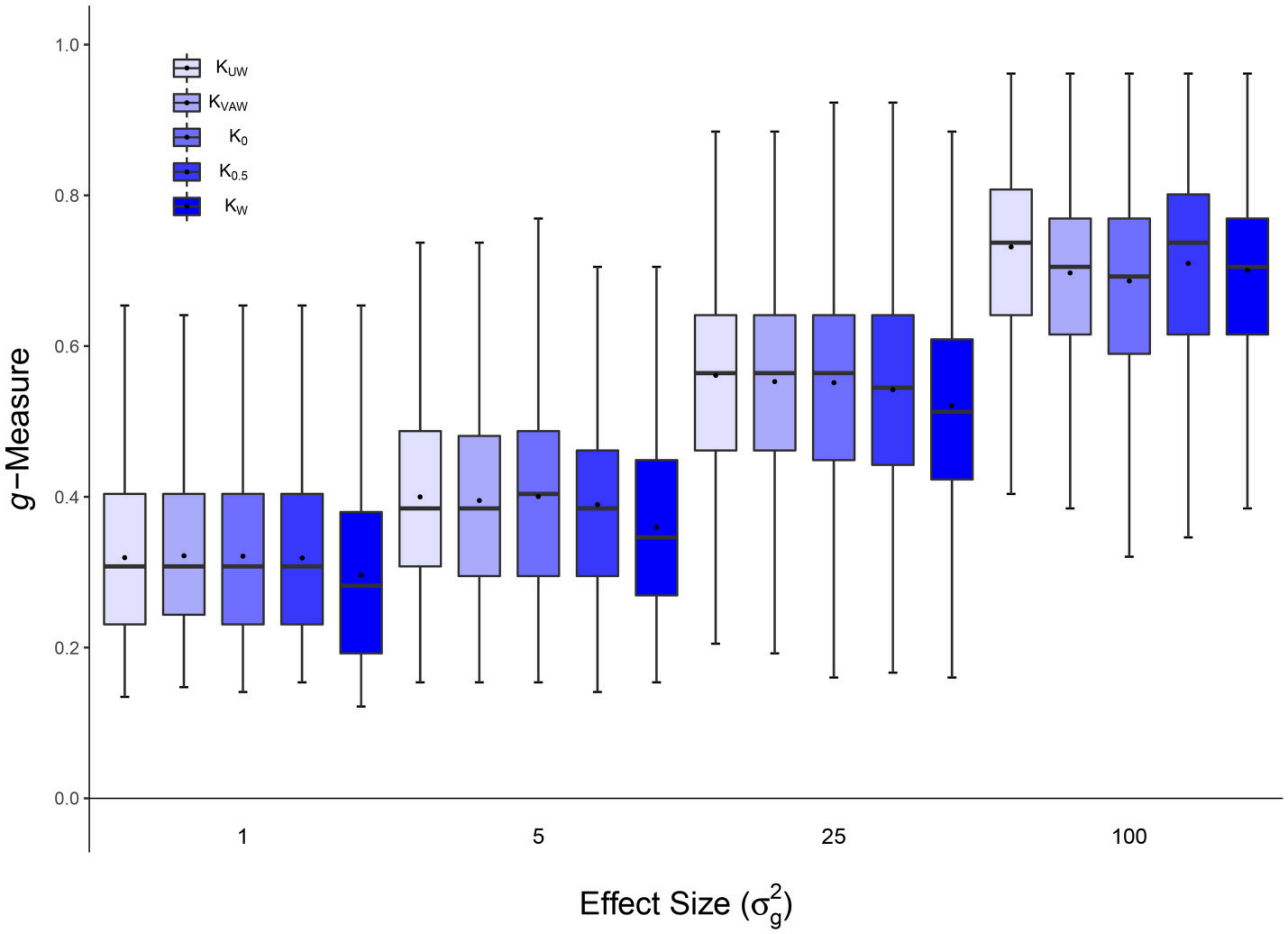
Scenario 3: Estimated ***g***-Measure of VC-lasso under different UniFrac distance kernels in a longitudinal design.Five different kernels, *K*_*UW*_, *K*_*VAW*_, *K*_0_, *K*_0.5_ and *K*_*W*_, are compared. There are 5 non-zero variance components. Sample size is *n* = 50 and $\sigma_{d}^{2}=0.6$.

**Figure 7:**
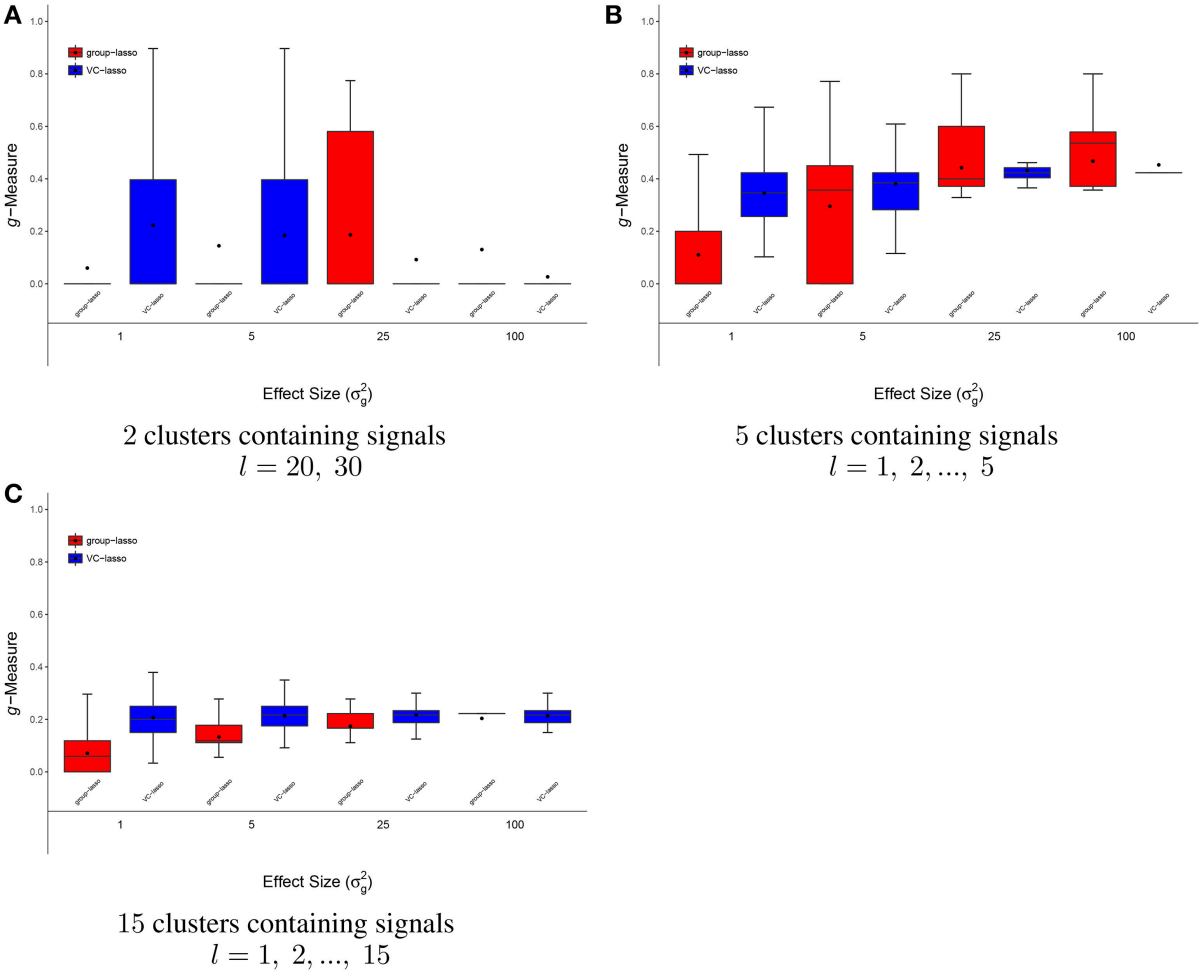
Scenario 4: Estimated ***g***-Measure of VC-lasso and group-lasso under fixed effect model in a cross-sectional design. There are 2 **(A)**, 5 **(B)**, 15 **(C)** clusters with signals. Sample size is *n* = 50 and $\sigma_{d}^{2}=0$.

**Figure 8:**
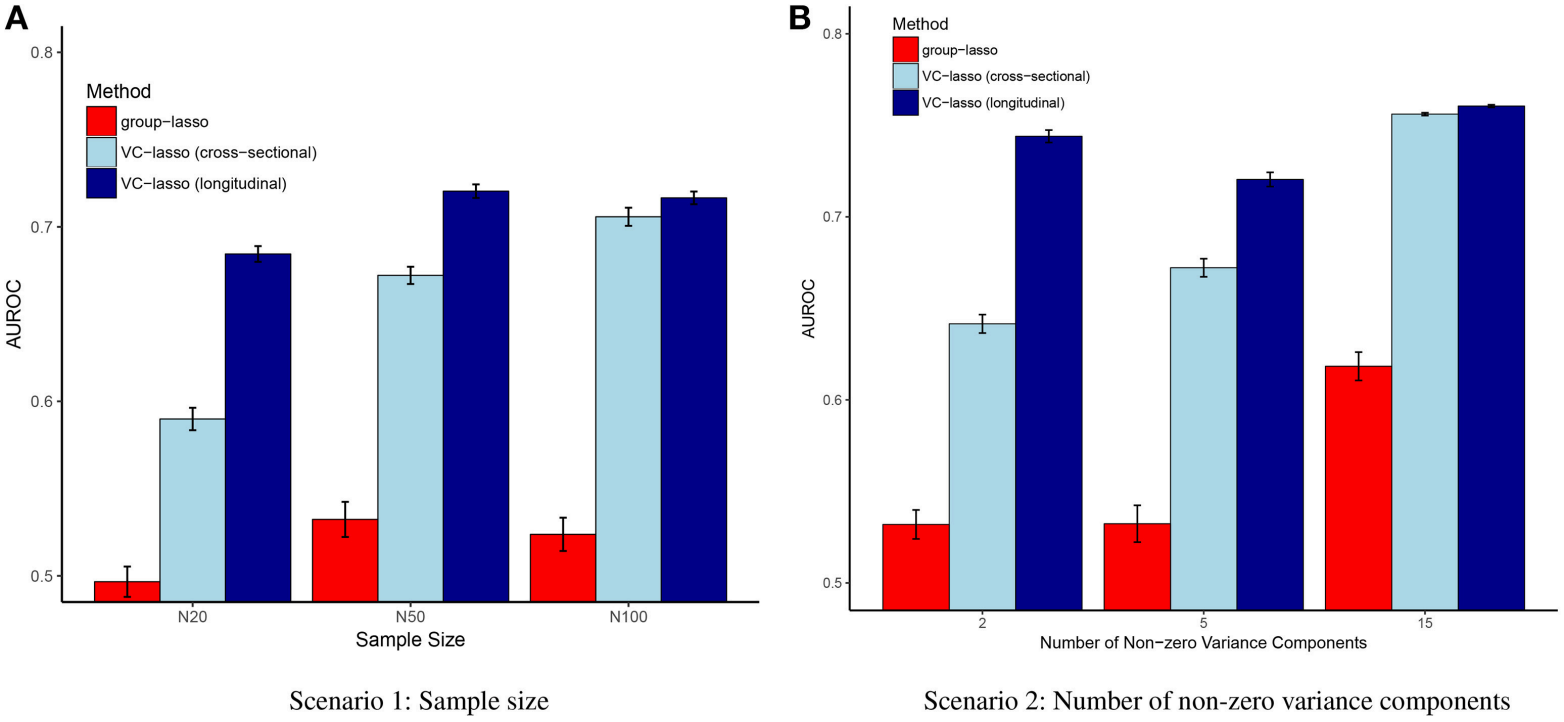
Scenario 1 & 2: AUROC. The AUROC is presented as the mean ± 95% confidence interval based on 1,000 simulation replicates for each simulation scenario when $\sigma_{g}^{2}=25$. **(A)** Scenario 1; **(B)** Scenario 2.

**Figure 9:**
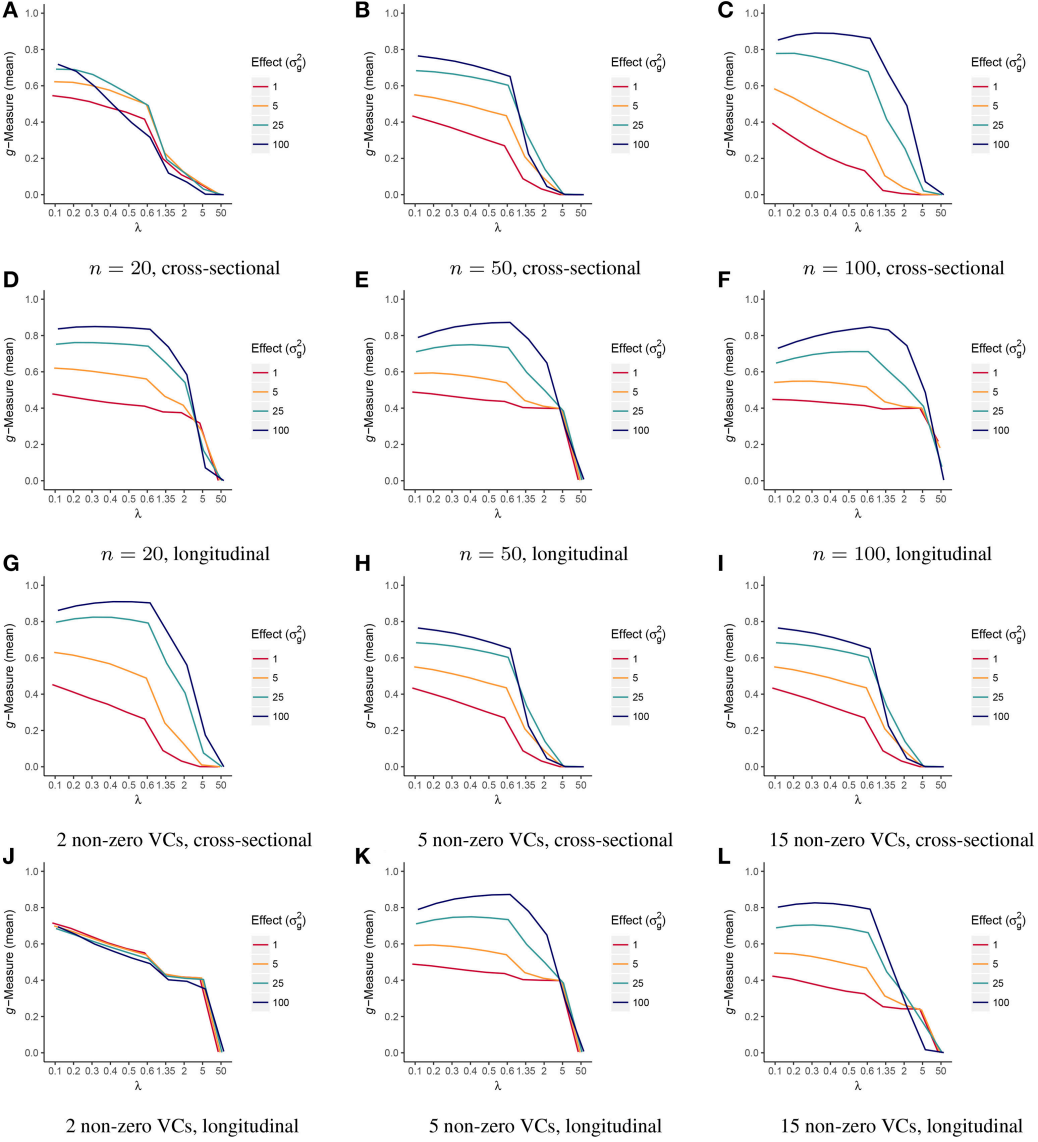
Trajectories of estimated ***g***-Measure as a function of tuning parameter **λ** (scenario 1 & 2). Estimated *g*-Measure is displayed as the mean of 5-fold cross-validation under sample sizes, *n* = 20 **(A,D)**, 50 **(B,E)**, 100 **(C,F)**, or 2 **(G,J)**, 5 **(H,K)**, 15 **(I,L)** non-zero variance components (VCs) in both cross-sectional and longitudinal designs.

The simulation results are summarized in Figures 1–9 including variable selection performance under different sample sizes (Figures 1, 2), different numbers of non-zero variance components (Figures 3, 4), and different UniFrac distance measures (Figures 5, 6) for both cross-sectional and longitudinal designs. Comparisons between VC-lasso and group-lasso are shown in all cross-sectional simulation studies.

The trajectories of *g*-Measure versus tuning parameter λ from cross-validation is presented in Figure 9. *g*-Measures remain stable or slightly decrease as λ getting larger under moderate effect size when $\sigma_{g}^{2}=1\text{and}\;5$. It starts to decrease when λ is greater than 0.6. Figure 9 suggests that the trajectories of tuning criteria is generally consistent across sample sizes, effect sizes, and study designs.

#### 4.1.1. Scenario 1: selection under different sample sizes

Figures 1, 2, 8A display performance of selection (*g*-Measure) and prediction (area under the receiver operating characteristic curve, AUROC). In Figure 1, we compare VC-lasso (blue bar) and group-lasso (red bar) using cross-sectional design. In Figure 2, we compare the *g*-Measure of VC-lasso under different sample sizes using a longitudinal design. For both cross-sectional and longitudinal designs, *g*-Measure of VC-lasso boosts with increased sample size and effect sizes. Except for the third quartile of *g*-Measure over 1,000 replicates for sample size, *n* = 20, VC-lasso always outperforms group-lasso in this scenario.

Area under receiver operating characteristic (AUROC) is used to evaluate the prediction performance (Figure 8A) when effect size is fixed at $\sigma_{g}^{2}=25$. Larger AUROC represents better prediction ability. For VC-lasso, AUROC increases with sample size under cross-sectional design. For longitudinal study, *n* = 50 has similar AUROC with *n* = 100, which indicates the optimal prediction we can receive under this simulation setting. The AUROC of group-lasso (red bar) is similar under different sample sizes and shows no advantages compared to the VC-lasso.

#### 4.1.2. Scenario 2: selection under different number of non-zero variance components

Figures 3, 4, 8B show simulation results for the selection under different number of non-zero variance components. Specifically, Figure 3 shows *g*-Measure for both VC-lasso and group-lasso in a cross-sectional design, while Figure 4 presents *g*-Measure for VC-lasso in a longitudinal design.

In Figures 3, 4, the performance of VC-lasso selection improves when effect size increases. For a model with 2 non-zero variance components, the true discovery rate (TDR, or sensitivity) is either 0, 0.5 or 1.0, which lead to a large variation of the *g*-Measure (Figure 3A). As more non-zero variance components are included, in Figure 3B,C the trajectory of *g*-Measures becomes smoother. The *g*-Measures of VC-lasso are higher than the group-lasso in most simulation settings except that group-lasso has larger third quartile when $\sigma_{g}^{2}=1$ in Figure 3A and $\sigma_{g}^{2}=5$ in Figure 3C. As shown in Figure 8B, VC-lasso has a better prediction ability with an increased number of non-zero variance components. Compared with our method, group-lasso is uncompetitive in predictive ability.

#### 4.1.3. Scenario 3: selection under different UniFrac distance kernels

We compare the *g*-Measure of five different kernels in Figures 5, 6 for the cross-sectional and longitudinal design, respectively. Using longitudinal simulated data, the box-plot of *g*-Measure shows that the five kernels have similar performance except that the *K*_*W*_ has the lowest third quartile and *K*_0_ has the lowest first quartile when $\sigma_{g}^{2}$ is large. Under the same effect strength ($\sigma_{g}^{2}$) in the cross-sectional design (Figure 5), the *g*-Measure of five kernels are almost identical except that *K*_0_ has slightly smaller *g*-Measure and wider range than other kernels. For example, *K*_0_ has the lowest first quartile in Figures 5B. This suggests that the kernels computed from different UniFrac distance play a minor part in the selection performance and our method is superior to group-lasso regardless of kernel types.

#### 4.1.4. Scenario 4: selection under fixed effect model

Figure 7 has a distinctive pattern from the above scenarios. For the case that only two microbiome clusters contain signals (*Prevotella* and *Veillonella*), both methods do not perform well (Figure 7A). In Figures 7B,C, *g*-Measures for both methods improve with increased effect sizes and VC-lasso outplays group-lasso with $\sigma_{g}^{2}=1$. For $\sigma_{g}^{2}=5,\;25$, average and median *g*-Measure of VC-lasso across simulation replicates outperform group-lasso. Besides, we notice that the range of *g*-Measure for VC-lasso becomes smaller as signal strengths increase, suggesting the prediction performance stabilizes as the association with the outcome increases. In general, VC-lasso has a distinctively better selection performance even when model is misspecified.

### 4.2. Application to longitudinal pulmonary microbiome data

We apply VC-lasso to a longitudinal dataset of pulmonary microbiome study. Bronchoalveolar lavage (BAL) fluid were collected for microbiome profiling. The inclusion criterion for this cohort were: (1) HIV infection and (2) CD4 count less than 500 *cells*/*mm*^3^ before HAART (Twigg III et al., 2016). Two most common pulmonary function tests were performed repeatedly: spirometry and diffusing capacity for carbon monoxide. In this report we focus on spirometry measures. Spirometry is to measure the lung volume and how well the lung exhales, such as average forced expiratory flow (FEF) and forced expiratory volume in 1s (FEV1). Both spirometry and diffusing capacity were evaluated as percent predicted values as pulmonary function tests are usually interpreted by comparing the patient's value to predicted value of the healthy subject with similar age, height and ethnicity (Twigg III et al., 2016).

Twigg III et al. (2016) compared microbiome abundance differences at overall community level between (1) uninfected and baseline; (2) uninfected and 1 year after treatment; and (3) uninfected and 3 year treated subjects. They suggest that the lung microbiome in healthy HIV-infected individuals with preserved CD4 counts is similar to uninfected individuals. Among individuals with more advanced disease, there is an altered alveolar microbiome characterized by a loss of richness and evenness (alpha diversity). This alteration might impact pulmonary complications (often characterized by the measure of lung functions) in HIV-infected patients on antiretroviral therapy (ART). In this application, we therefore aim to identify microbiome genera associated with pulmonary function in both longitudinal and baseline studies. Ethnicity, gender, smoking history, CD4 count, and HIV viral load are included as the covariates. Missing covariates are imputed by their mean. Penalized variance component selection is performed among all 31 genera. Due to limited sample sizes, we choose the optimal tuning parameter λ^*^ by AIC and BIC.

**Figure 10:**
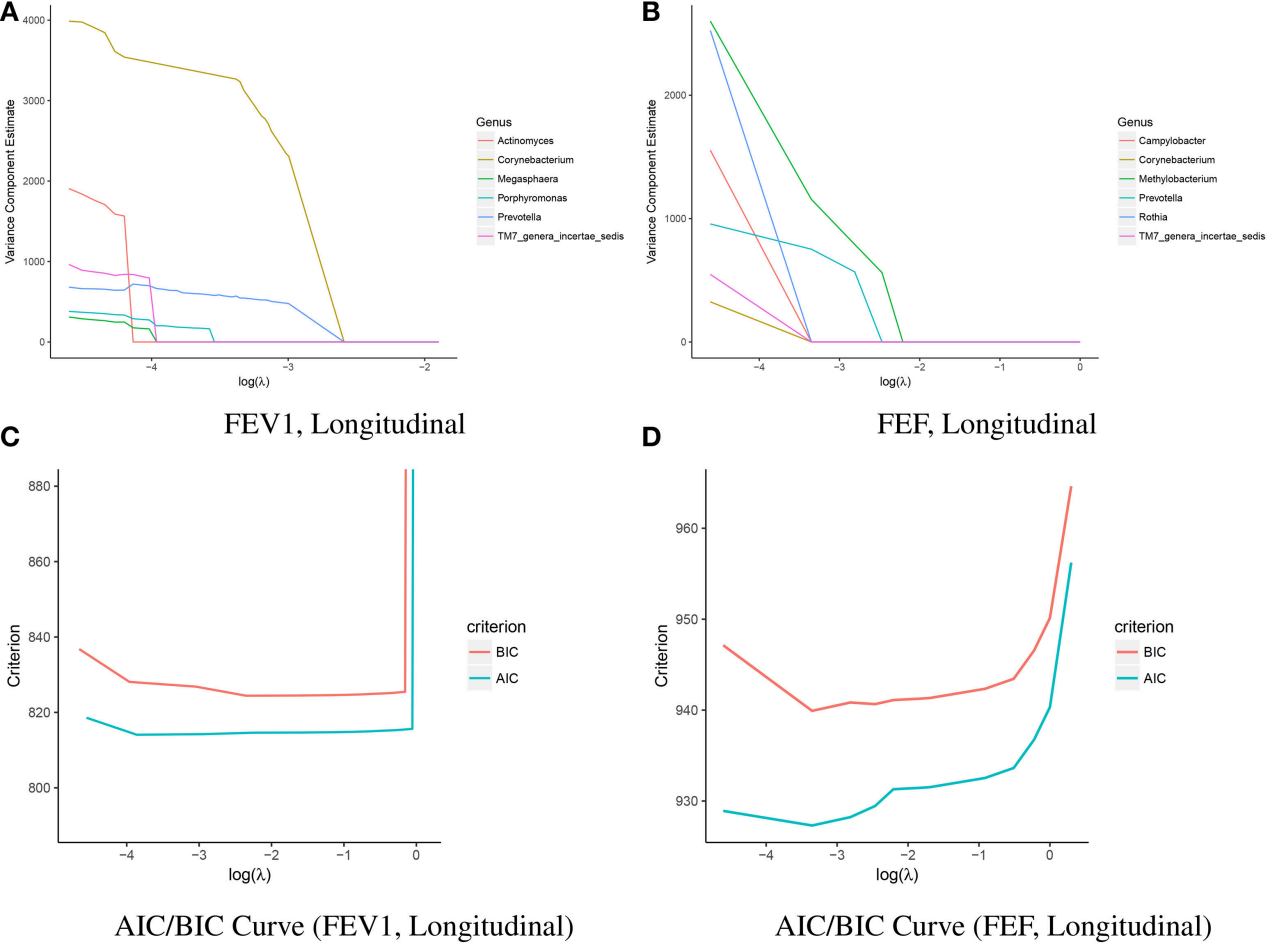
Solution path and AIC/BIC curve of the VC-lasso method in the analysis of 31 genera and the pulmonary function. The solution paths with penalty parameter are presented for FEV1 **(A)** and FEF **(B)** in longitudinal study (upper panel). AIC/BIC curves as a function of tuning parameter for FEV1 **(C)** and FEF **(D)** are shown in the lower panel.

Tables 3, 4 show selected genera with their phylum information and the corresponding *p*-values from exact tests, i.e., score test (eScore), likelihood ratio test (eLRT), and restricted likelihood ratio test (eRLRT) (Zhai et al., 2017b). The genera are ranked in the order they appear in the solution path (Figures 10A,B). VC-lasso selects 6 genera associated with FEV1 using longitudinal data and λ^*^ = 0.2 (Table 3 and Figure 10C). Three out of six selected genera have eRLRT *p*-values < 0.05 (Table 3), including *Actinomyces* (*p* < 0.01), *Prevotella* (*p* = 0.01), and *Porphyromonas* (*p* < 0.01). Using baseline data, we identify five genera associated with FEV1, among which *Corynebacterium* and *TM7 genera incertae sedis* are also selected by using longitudinal data. Several selected genera received insufficient attention in HIV-infected populations previously, for example, *Anaerococcus* and *Megasphaera*. Studies have shown that *Anaerococcus* became more abundant in children with asthma after azithromycin treatment (Slater et al., 2013; Riiser, 2015) and *Megasphaera* has higher relative abundance in smoking population (Segal et al., 2014). However, none of them has been reported in HIV infected pulmonary microbiome (Rogers et al., 2004; Chen et al., 2007; Twigg III et al., 2016).

**Table 3:** Analysis of Forced expiratory volume in one second (FEV1) at genus level in the real pulmonary microbiome cohort using variance component lasso selection (VC-lasso) and exact tests.

	**VC-lasso**			**Exact tests**		
	**Rank**	**Genus**	**Phylum info**	**eRLRT**	**eLRT**	**eScore**
Baseline	1	*Corynebacterium*	*Actinobacteria*	0.28	0.30	0.30
	2	*TM7_genera_incertae_sedis*	*TM7*	1.00	1.00	1.00
	3	*Anaerococcus*	*Firmicutes*	0.06	0.06	0.07
	4	*Neisseria*	*Proteobacteria*	1.00	1.00	1.00
	5	*Treponema*	*Spirochaetes*	0.13	0.14	0.14
Longitudinal	1	*Corynebacterium*	*Actinobacteria*	1.00	–	1.00
	2	*Actinomyces*	*Actinobacteria*	0.00	–	0.01
	3	*Prevotella*	*Bacteroidetes*	0.01	–	0.01
	4	*TM7_genera_incertae_sedis*	*TM7*	1.00	–	1.00
	5	*Porphyromonas*	*Bacteroidetes*	0.00	–	0.00
	6	*Megasphaera*	*Firmicutes*	0.06	–	0.06

The phylum information is provided for selected genera. Tuning parameter λ* for baseline and longitudinal data is set to 0.01 and 0.2, respectively. Rank represents the order of genera that appear in the solution path. Results of eLRT are omitted as it is equivalent to eRLRT in a longitudinal design.

**Table 4:** Analysis of forced expiratory flow (FEF) at genus level in the real pulmonary microbiome cohort using variance component lasso selection (VC-lasso) and exact tests.

	**VC-lasso**			**Exact tests**		
	**Rank**	**Genus**	**Phylum info**	**eRLRT**	**eLRT**	**eScore**
Baseline		–	–	–	–	–
Longitudinal	1	Methylobacterium	Proteobacteria	1.00	–	1.00
	4	Prevotella	Bacteroidetes	<0.01	–	<0.01
	2	Rothia	Actinobacteria	0.01	–	0.03
	3	Campylobacter	Proteobacteria	0.03	–	0.03
	5	TM7_genera_incertae_sedis	TM7	0.00	–	0.01
	6	Corynebacterium	Actinobacteria	0.32	–	0.31

The phylum information is provided for selected genus. Tuning parameter λ^*^ = 0.035 for longitudinal data. Rank represents the order of genera that appear in the solution path. No genus is chosen using baseline data only. Results of eLRT are omitted in longitudinal design as it is equivalent to eRLRT.

For variance component selection on FEF (Table 4), VC-lasso selects 6 genera in total using longitudinal data with λ^*^ = 0.035. Considering the exact test results (eRLRT and eScore), four of them show significant association with FEF (*p*-value < 0.05), i.e., *Prevotella, Rothia, Campylobacter* and *TM7_genera_incertae_sedis*. Twigg III et al. (2016) reported that HIV-positive BAL samples contained an increased abundance of *Prevotella* after 1-year HAART treatment while significantly decreased abundances during 3 years of treatment. *Campylobacter* is another noteworthy genus that has significant association with inflammation markers of HIV-infected population (Iwai et al., 2014). Additionally, significantly increased abundance of *Rothia* and *TM7_genera_incertae_sedis* in oral wash microbiome has been reported in HAART treatment group (Iwai et al., 2012; Beck et al., 2015). In conclusion, VC-lasso provides innovative association evidence between fine level pulmonary microbiome clusters with lung function phenotypes. Our report is a hypothesis generation procedure. Association results need to be further validated in a separate population or by laboratory experiments.

## 5. Discussion

In this paper, we propose the variance component selection scheme VC-lasso for sparse and high-dimensional taxonomic data analysis. To reduce the dimensionality, we first aggregate the dispersed individual OTUs to clusters at higher phylogenetic level, such as genus, family, or phylum. By translating the phylogenetic distance information to kernel matrices, we treat the aggregated taxonomic clusters as multiple random effects in a variance component model. Then, VC-lasso is performed for parsimonious variable selection of variance components. The MM algorithm with lasso penalization derived in Algorithm 1 for parameter estimation extremely simple and computationally efficient for variance component estimation. The group-lasso as a comparison can also be used for the microbiome cluster selection and incorporating higher phylogenetic group information (Yuan and Lin, 2006; Garcia et al., 2013; Yang and Zou, 2015). However, group-lasso suffers from the high-dimensionality and sparsity of OTUs within clusters. And group-lasso is not easy to accommodate phylogenic information. Beyond that, our novel approach VC-lasso can be applied to longitudinal designs. In such cases, we do not penalize the variance component that contains the repeat measurement correlation. Software and detailed documentation are freely available at https://github.com/JingZhai63/VCselection.

The VC-lasso is not limited to random intercept model for longitudinal studies. More complex random effect models, such as random intercept and random slope model, can also be used. More generally, the extension of our method to multivariate responses is expected to have better prediction performances. In the precision medicine era, with the rapid development of sequencing techniques and decreasing costs, the personal microbiome sequencing is already available to the consumer, e.g., American Gut (http://americangut.org/) and uBiome (https://ubiome.com/). Selection for higher-order interactions with random effect, such as microbiome and treatment regime interactions (Gopalakrishnan et al., 2017), will be a straightforward, yet interesting, implementation (Maity and Lin, 2011; Lin et al., 2016).

In practice, knowledge is needed about which taxonomy level should be aimed at to develop strategies for intervention. Considering multiple level taxonomic data, one can extend VC-lasso to include tree topologies (Wang and Zhao, 2016; Wang et al., 2017). For example, overlapping or subgroup VC-lasso can be developed by using both ℓ_1_ and ℓ_2_ regularizations (Jacob et al., 2009; Bien et al., 2013). Last but not the least, the variance components model requires specification of a kernel function or kernel matrix a priori, but it is often unclear which distance kernel to use in practice. To deal with the uncertainty, we can consider obtaining a composite kernel by utilizing a multiple kernel learning algorithm, such as a multi-kernel boosting algorithm (Xia and Hoi, 2013). In conclusion, with its competitive performance and many potential extensions, our variance components model with regularization, VC-lasso, is a powerful tool for mining the emerging microbiome data.

## Author contributions

JZ implemented method and carried out data analysis. JZ wrote the manuscript with support from JK, HZ, and JJZ. HZ helped supervise the project. HT and KK provided pulmonary microbiome data. JJZ supervised the project.

### Conflict of interest statement

The authors declare that the research was conducted in the absence of any commercial or financial relationships that could be construed as a potential conflict of interest.
