# Supplementary material for: Variance Component Selection With Applications to Microbiome Taxonomic Data
Source: Front Microbiol. 2018 Mar 28;9:509. doi: 10.3389/fmicb.2018.00509 (PMC5883493; doi:10.3389/fmicb.2018.00509)
Supplement: Supplementary file 1 [file DataSheet1.pdf]

Supplementary Material: Variance Component Selection with Microbiome Taxonomic Data

Jing Zhai [1], Juhyun Kim [4], Kenneth Knox [2], Homer Twigg [3], Hua Zhou [4], Jin J. Zhou [1,*]
*Correspondence:
Jin J. Zhou
jzhou@email.arizona.edu

1 UNIFRAC DISTANCE KERNEL

For each individual i we get n_i repeated microbiome profile measurements. Consider two microbiome communities A and B and suppose that we have a rooted phylogenetic tree with m branches(m is the total number of OTUs). Let b_i denote the length of branch i ($i = 1, 2, \cdots, m$) and p_i^A, p_i^B are the taxa proportions descending from the branch i for community A and B respectively. T is the total reads of m OTUs and T_i is total reads of ith OTU from both community.
Unweighted Unifrac (Lozupone and Knight, 2005)

$$d^U = \sum_{i=1}^{m} \frac{b_i |I(p_i^A > 0) - I(p_i^B > 0)|}{\sum_{i=1}^{m} b_i}$$

Weighted Unifrac (Lozupone et al., 2007)

$$d^W = \frac{\sum_{i=1}^{m} b_i |p_i^A - p_i^B|}{\sum_{i=1}^{m} b_i (p_i^A - p_i^B)}$$

VAW Unifrac (Chang et al., 2011)

$$d^{VAW} = \frac{\sum_{i=1}^{m} b_i \dfrac{|p_i^A - p_i^B|}{T(T - T_i)}}{\sum_{i=1}^{m} b_i \dfrac{|p_i^A - p_i^B|}{T(T - T_i)}}$$

Generalized Unifrac (Chen et al., 2012)

$$d^{(\alpha)} = \frac{\sum_{i=1}^{m} b_i (p_i^A + p_i^B)^\alpha |\dfrac{p_i^A - p_i^B}{p_i^A + p_i^B}|}{\sum_{i=1}^{m} b_i (p_i^A + p_i^B)^\alpha}$$

We define a kernel matrices by transforming the distance matrices through equation S1 to measure similarities between the microbiome composition among subjects.

$$\boldsymbol{K} \quad = \quad -\frac{1}{2}(\boldsymbol{I} - \frac{\mathbf{11}'}{N})\boldsymbol{D}^2(\boldsymbol{I} - \frac{\mathbf{11}'}{N}) \tag{S1}$$

where $\boldsymbol{D} = (d_{ii'})$ is the pairwise distance matrice, $\boldsymbol{I}$ is the identity matrice and $\mathbf{1}$ is a vector of $\mathbf{1}$'s. The N denotes the total number of samples.

2 MICROBIOME COUNT SIMULATION

The microbiome count data measured at multiple time points are expected to be correlated in longitudinal study. We adopt the two-part zero-inflated Beta regression model with random effects proposed by Chen and Li (2016) to simulate correlated microbiome count data. The ZIBR model considers each bacterial taxon separately. We illustrate the simulation procedure using one taxon as example:
Step 1. Given the real pulmonary microbiome dataset, we first compute the mean reads for each bacterial taxon, e.g., OTU_u, $u = 1,\ 2,\ ...,\ 2964$.
Step 2. We first simulate the absence/presence of OTU_u in ZIBR model's logistic component S2,

$$logit(p_{it}) = log\left(\frac{p_{it}}{1 - p_{it}}\right) = \alpha_0 + treatment_{it} * \alpha_1 + time_{it} * \alpha_2 + a_i \tag{S2}$$

where $treatment$ is a binary covariate, $treatment = 0$ for $time = 0$ and $treatment = 1$ for $time > 0$. p_{it} is the probability of OTU_u present in the subject i at time t.
Step 3. For the present OTU_u, we then simulate its abundance, namely the proportion of overall taxon reads for subject i. The abundance follow the Beta distribution with mean μ_{it} and dispersion ϕ. The μ_{it} depend on the $treatment$ and $time$ through the logit link function S3

$$logit(\mu_{it}) = log\left(\frac{\mu_{it}}{1 - \mu_{it}}\right) = \beta_0 + treatment_{it} * \beta_1 + time_{it} * \beta_2 + b_i \tag{S3}$$

where a_i and b_i is the random effect in the two part logistic model to account for the correlation of repeated measurement.
Step 4. The ZIBR model return the proportion instead of reads for each bacterial taxon, the simulated microbiome counts are obtained by multiplying the proportion with mean reads from step 1.

3 FALSE DISCOVERY RATE AND TRUE POSITIVE RATE OF VC-LASSO

In Figure S1 and Figure S2, we evaluate VC-lasso using false discovery rate (FDR) and true positive rate (TPR) by equation S4. FDR of VC-lasso in both simulation scenario 1 and scenario 2 are in reasonable range. FDR decreases with the number of true variance components decrease. For example, FDR for the model with 2 non-zero variance components is lower than the model with 15 non-zero variance components. TPR increases when the effect sizes increase as shown in Figure S1-Figure S2

$$\text{FDR} = \frac{\text{false positive}}{\text{false positive} + \text{true positive}};\ \text{TPR} = \frac{\text{true positive}}{\text{true positive} + \text{false negative}} \tag{S4}$$

4 AKAIKE INFORMATION CRITERION AND BAYESIAN INFORMATION CRITERION

In Figure S3-Figure S4, we show the performance of VC-lasso when optimal tuning parameter λ is selected by Akaike Information Criterion (AIC) and Schwarz's Bayesian Information Criterion (BIC). Specifically, Figure S3 show variable selection performance under different sample sizes while Figure S4 show variable selection performance under different number of non-zero variance components. Compare to the cross-validation, AIC and BIC procedures lead to slightly higher g-Measure under sample size $n = 20$ and effect size greater than 5. When sample sizes are $50,\ 100$, cross-validation perform better. Cross-validation is also better with less non-zero variance components exist. Although we suggest cross-validation should be used to choose tuning parameter, the performance of AIC and BIC are comparable to cross-validation when sample sizes are limited.

5 MODELS WITH LARGER NUMBER OF VARIANCE COMPONENTS

In this supplementary simulation, we aim to evaluate the performance of VC-lasso when the number of variance components in the model is large, for example, $L = 100$. We randomly group 998 non-zero OTUs into 100 clusters. The maximum of OTUs in each clusters is 18 and the minimum is 4. We fix sample size at $n = 50$ and responses are simulated when $\sigma_d^2 = 0$. The true model has five non-zero variance components. As shown in Figure S5, VC-lasso works well in large scale variance components models even with 100 clusters. VC-lasso is also very efficient. It took only 11.9 seconds to run one simulation replicate using five fold cross-validation on a grid of 11 values for the regularization parameter λ. The simulation was run on High Performance Computing (HPC) cluster using 1 node, 28 cores, and 168GB RAM in total. This cluster uses Intel Haswell V3 28 core processors with 192GB RAM per node.

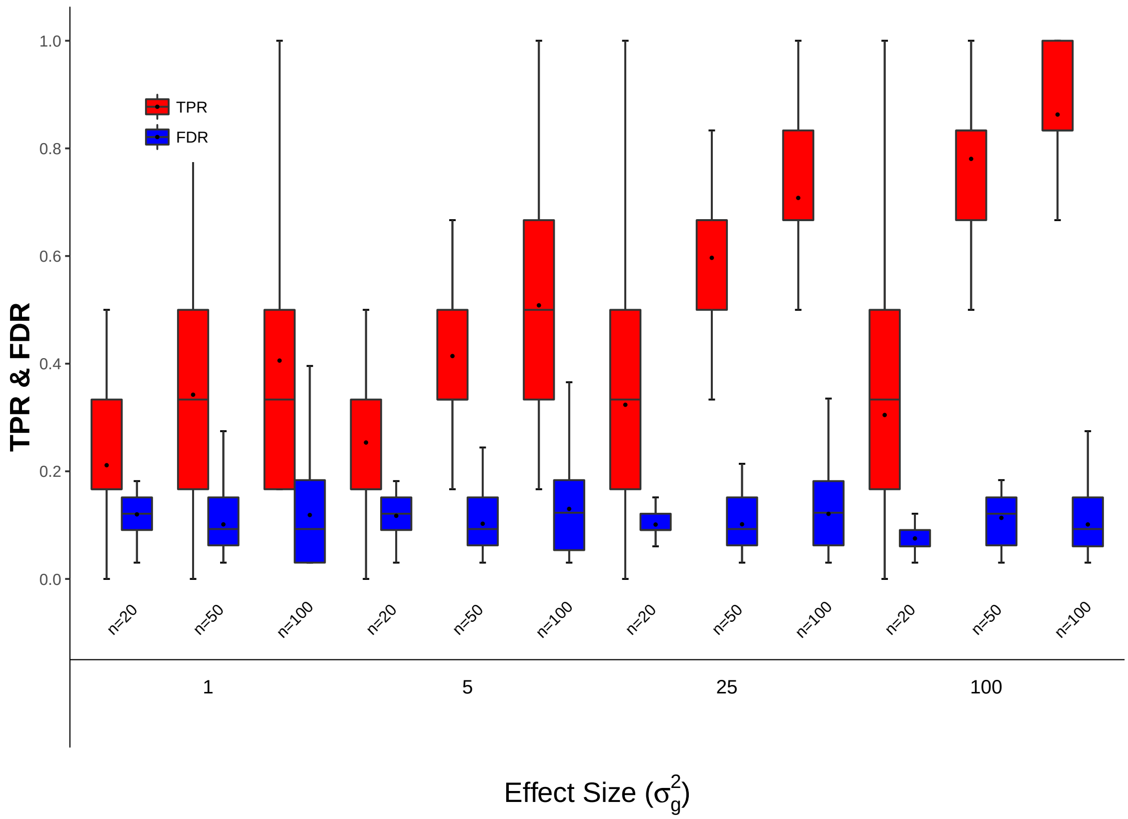

Figure S1: **Scenario 1: Estimated FDR and TPR of VC-lasso under different sample sizes for models with 5 non-zero variance components in a longitudinal design.** Three sample sizes, $n = 20,\ 50,\ 100$, are compared and $\sigma_d^2 = 0.6$.

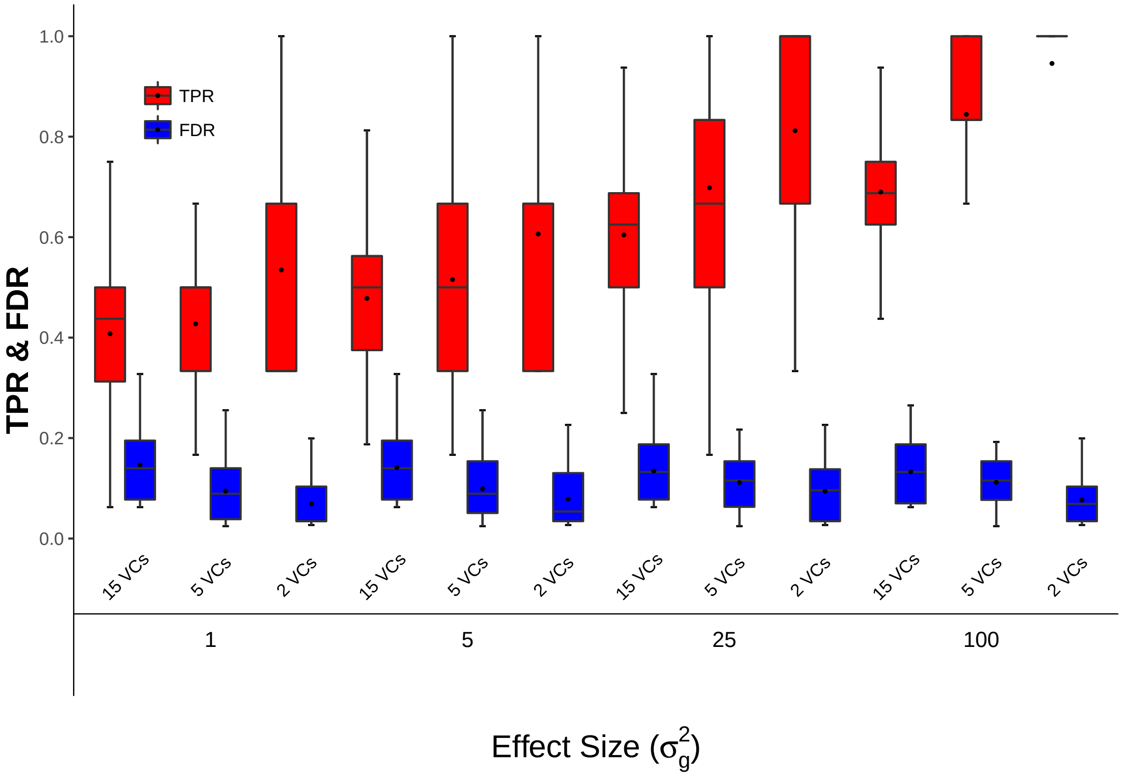

Figure S2: **Scenario 2: Estimated FDR and TPR of VC-lasso under different number of non-zero variance components in a longitudinal design.** Three different numbers of non-zero variance components, $2,\ 5,\ 15$, are shown, sample size is set to $n = 50$ and $\sigma_d^2 = 0.6$.

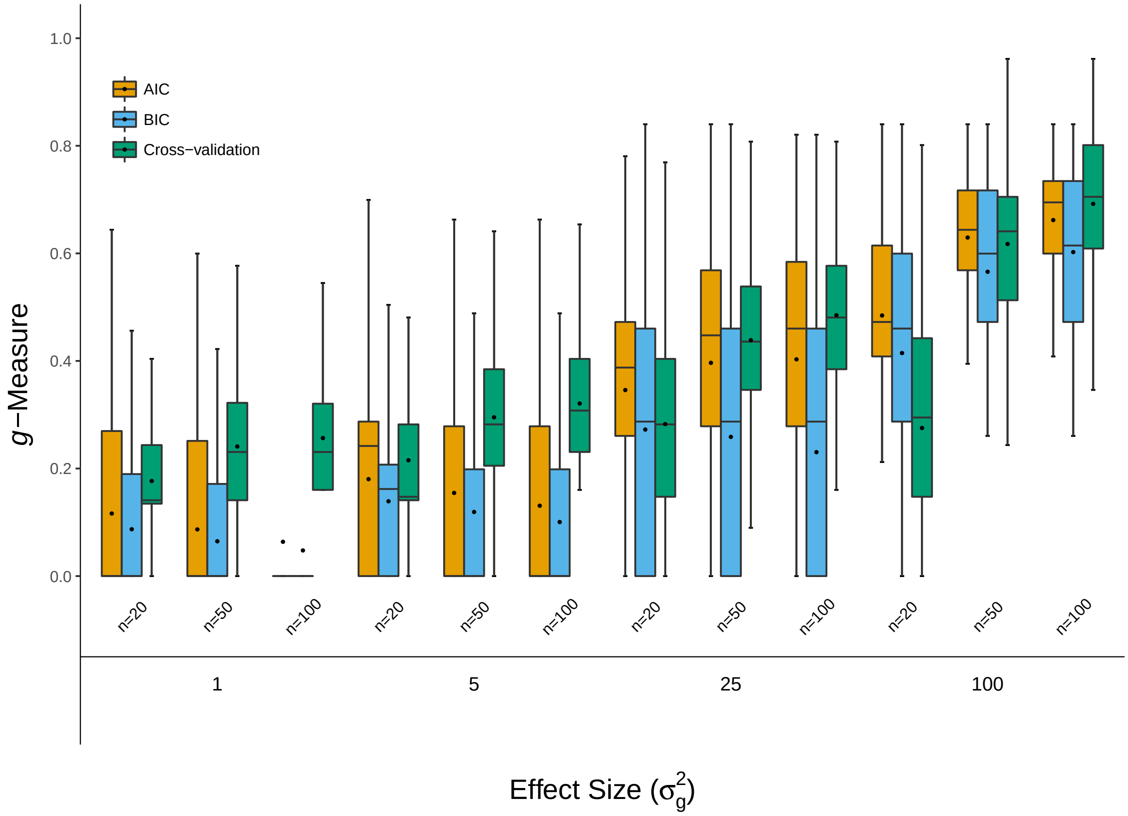

Figure S3: **Scenario 1: Estimated g-Measure of VC-lasso (AIC, BIC and Cross-validation) under different sample sizes for models with 5 non-zero variance components in a longitudinal design.** Three sample sizes, $n = 20,\ 50,\ 100$, are compared and $\sigma_d^2 = 0.6$.

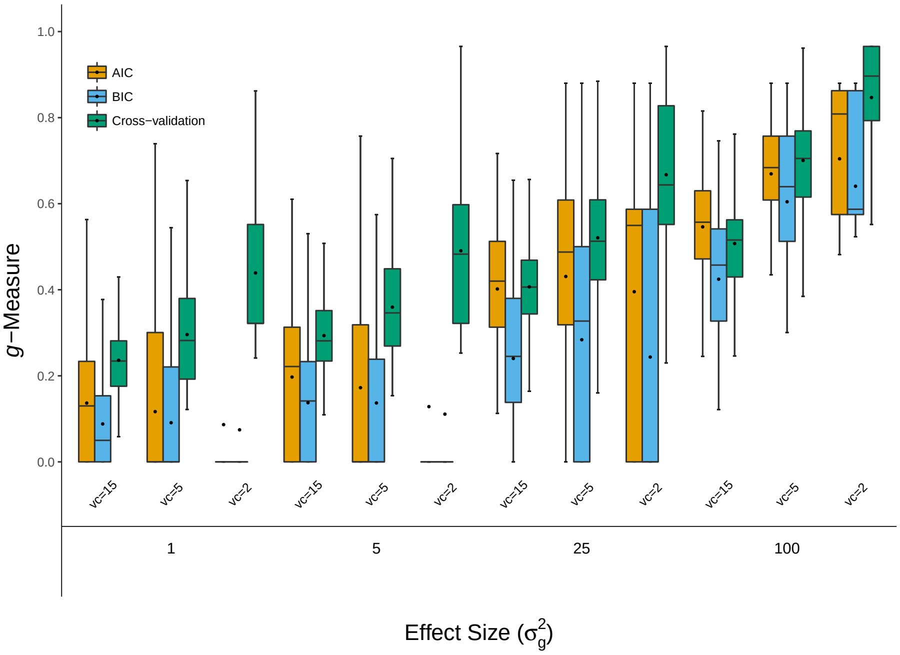

Figure S4: **Scenario 2: Estimated g-Measure of VC-lasso (AIC, BIC and Cross-validation) under different number of non-zero variance components in a longitudinal design.** Three different numbers of non-zero variance components, $2,\ 5,\ 15$, are shown, sample size is set to $n = 50$ and $\sigma_d^2 = 0.6$.

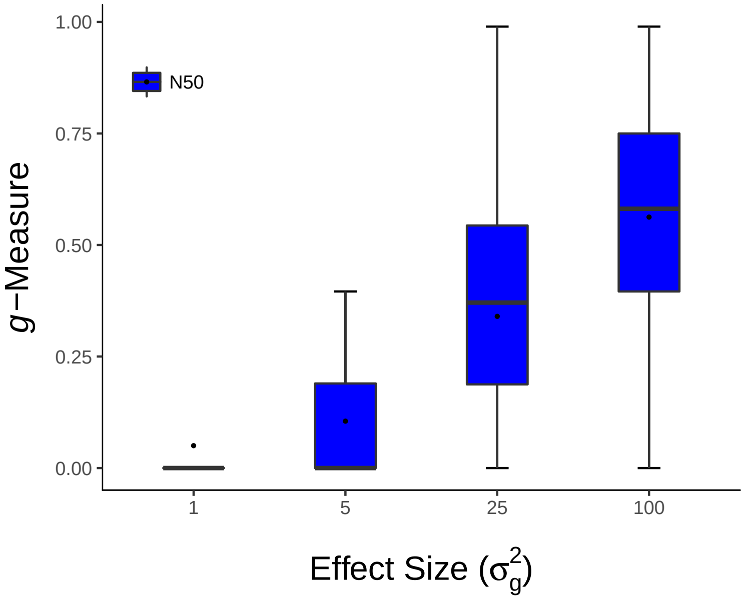

Figure S5: **Estimated g-Measure of VC-lasso with 5 non-zero and 95 zero variance components using cross-sectional design.** The 998 OTUs are randomly aggregate to 100 clusters with maximum 18 OTUs and minimum 4 OTUs in one cluster. Responses are simulated with 5 true variance components in 100 clusters. Sample size is set to $n = 50$ and $\sigma_d^2 = 0$.

REFERENCES

Chang, Q., Luan, Y., and Sun, F. (2011). Variance adjusted weighted unifrac: a powerful beta diversity measure for comparing communities based on phylogeny. *BMC bioinformatics* 12, 118

Chen, E. Z. and Li, H. (2016). A two-part mixed-effects model for analyzing longitudinal microbiome compositional data. *Bioinformatics* 32, 2611–2617. doi:10.1093/bioinformatics/btw308

Chen, J., Bittinger, K., Charlson, E. S., Hoffmann, C., Lewis, J., Wu, G. D., et al. (2012). Associating microbiome composition with environmental covariates using generalized unifrac distances. *Bioinformatics* 28, 2106–2113

Lozupone, C. and Knight, R. (2005). Unifrac: a new phylogenetic method for comparing microbial communities. *Applied and environmental microbiology* 71, 8228–8235

Lozupone, C. A., Hamady, M., Kelley, S. T., and Knight, R. (2007). Quantitative and qualitative β diversity measures lead to different insights into factors that structure microbial communities. *Applied and environmental microbiology* 73, 1576–1585
